# Genomic tools for durum wheat breeding: de novo assembly of Svevo transcriptome and SNP discovery in elite germplasm

**DOI:** 10.1186/s12864-019-5645-x

**Published:** 2019-04-10

**Authors:** Vera Vendramin, Danara Ormanbekova, Simone Scalabrin, Davide Scaglione, Marco Maccaferri, Pierluigi Martelli, Silvio Salvi, Irena Jurman, Rita Casadio, Federica Cattonaro, Roberto Tuberosa, Andrea Massi, Michele Morgante

**Affiliations:** 1 IGA Technology Services, via J. Linussio 51, 33100 Udine, Italy; 2 Department of Agricultural and Food Sciences DISTAL, University of Bologna, Viale G. Fanin 44, 40127 Bologna, Italy; 3 Biocomputing Group, University of Bologna, via San Giacomo 9/2, 40126 Bologna, Italy; 4 Istituto di Genomica Applicata, via J. Linussio 51, 33100 Udine, Italy; 5 Società produttori Sementi Bologna, Via Macero 1, 40050 Argelato, BO Italy; 6 Department od Agricultural, Food, Environmental and Animal Research - DI4A, University of Udine, via delle Scienze 206, 33100 Udine, Italy

**Keywords:** Durum wheat, *Triticum turgidum durum*, Transcriptome, de novo assembly, SNPs, Homeologs

## Abstract

**Background:**

The tetraploid durum wheat (*Triticum turgidum* L. ssp. *durum* Desf. Husnot) is an important crop which provides the raw material for pasta production and a valuable source of genetic diversity for breeding hexaploid wheat (*Triticum aestivum* L.). Future breeding efforts to enhance yield potential and climate resilience will increasingly rely on genomics-based approaches to identify and select beneficial alleles. A deeper characterisation of the molecular and functional diversity of the durum wheat transcriptome will be instrumental to more effectively harness its genetic diversity.

**Results:**

We report on the de novo transcriptome assembly of durum wheat cultivar ‘Svevo’. The transcriptome of four tissues/organs (shoots and roots at the seedling stage, reproductive organs and developing grains) was assembled de novo, yielding 180,108 contigs, with a N50 length of 1121 bp and mean contig length of 883 bp. Alignment against the transcriptome of nine plant species identified 43% of transcripts with homology to at least one reference transcriptome. The functional annotation was completed by means of a combination of complementary software. The presence of differential expression between the A- and B-homoeolog copies of the durum wheat tetraploid genome was ascertained by phase reconstruction of polymorphic sites based on the *T. urartu* transcripts and inferring homoeolog-specific sequences. We observed greater expression divergence between A and B homoeologs in grains rather than in leaves and roots. The transcriptomes of 13 durum wheat cultivars spanning the breeding period from 1969 to 2005 were analysed for SNP diversity, leading to 95,358 non-rare, hemi-SNPs shared among two or more cultivars and 33,747 locus-specific (diploid inheritance) SNPs.

**Conclusions:**

Our study updates and expands the de novo transcriptome reference assembly available for durum wheat. Out of 180,108 assembled transcripts, 13,636 were specific to the Svevo cultivar as compared to the only other reference transcriptome available for durum, thus contributing to the identification of the tetraploid wheat pan-transcriptome. Additionally, the analysis of 13 historically relevant hallmark varieties produced a SNP dataset that could successfully validate the genotyping in tetraploid wheat and provide a valuable resource for genomics-assisted breeding of both tetraploid and hexaploid wheats.

**Electronic supplementary material:**

The online version of this article (10.1186/s12864-019-5645-x) contains supplementary material, which is available to authorized users.

## Background

Durum wheat (*Triticum turgidum* L. ssp. *durum* Desf. Husnot) is a naked, free-threshing domesticated tetraploid wheat derived from a natural intergeneric hybridisation and polyploidisation event involving the A and B genomes of *Triticum urartu* and an unknown species related to *Aegilops speltoides* [1]. It is believed that the diploid progenitors of wheat diverged from a common ancestor about 2.5–4.5 million years ago [2], which would explain the relatively high sequence identity of coding regions among the different wheat homoeologs [3]. Wild emmer (*T. turgidum* ssp. *dicoccoides* Körn) was domesticated as emmer wheat (*Triticum turgidum ssp. dicoccum* Schrank) approximately 10,000 years ago and subsequently as durum wheat, hence undergoing successive reductions in population size and genetic diversity [4]. Compared to diploid wheats (einkorn), tetraploid wheats were more attractive for domestication due to a larger ear and seed size.

Assembling tetraploid wheat sequences poses significant challenges owing to the large genome size and its high redundancy [5, 6] as well as the high level of chromosomal rearrangements [7]. Therefore, the conservation of gene content and expression patterns across species is unknown. For the above reasons, de novo assembly of the transcriptome is essential for the identification of candidate genes, the development of SNP markers and genomic analyses. Moreover, particularly important is the correct identification, hence separation, of the homoeolog sequences.

A remarkable advancement in high-throughput sequencing of transcriptomes in polyploid species was achieved by adopting a multiple *k-mer* assembly strategy that allowed Krasileva et al. [8] to obtain a high-quality transcriptome assembly of the durum wheat cultivar Kronos. The same authors separated homoeolog sequences as reported also in previous work [7, 9] along with the development of a specific tool to disentangle homoeolog contigs in durum wheat genes [10]. The final transcriptome resulted in 140,118 contigs of *T. turgidum* and 66,633 predicted ORFs that were functionally annotated using a comparative genomics approach. An evaluation of the assembly showed that 96% of a benchmark full-length cDNA dataset [11] is assembled in a single contig.

In 2014, the International Wheat Genome Sequencing Consortium (IWGSC) released a reference wheat genome exploiting flow cytometry to isolate, sequence and assemble chromosome arms individually [12]. Recently, 3D chromosome-conformation capture coupled with high-throughput sequencing (Hi-C) was adopted to generate a high-quality reference genome sequence of the barley cultivar Morex [13] and a wild emmer wheat accession Zavitan [14].

Herein we report a de novo assembly of the tetraploid wheat transcriptome of cultivar Svevo as a complement to the reference transcriptome from cv. Kronos made available in 2013. In addition, we describe a large set of intervarietal SNPs derived from the sequencing of 13 elite durum cultivars (Additional file 1: Table S1) which are useful for marker-assisted breeding purposes. Finally, we developed a novel pipeline to generate homoeolog-specific information while using multiple approaches to annotate the transcriptome, hence providing deep gene functional information.

## Results

### De novo assembly and validation

A total of 384 million paired reads of cv. Svevo, distributed among the four tissues (see Methods), were generated (Additional file 2: Table S2 reports fragment size, number of reads and sequenced bases, average sequence length after trimming). Out of 384 million reads, 14 million (3.6%) were either removed based on their match with *E. coli* or mitochondrial and chloroplastic genomes or quality-trimmed from both ends. A final amount of 370 million reads were retained for transcriptome analysis.

To evaluate and choose the best performing de novo transcriptome assembly, a total of 20 and 25 assemblies were produced using CLC-Genomics Workbench v5.1 and Velvet/Oases, respectively (see Methods). The CLC software version used was able to perform automated clustering of contigs. Using the cleaned reads from either single tissues or all tissues together, assemblies with different *k-mer* sizes were performed. A detailed summary of the de novo assemblies is described in Additional file 3: Table S3). In CLC, the use of the longest available *k-mer* size (k = 64 bp) provided the best performance in terms of contig length, with values close to the expected length distribution for transcripts/genes (Additional file 4: Figure S1). With the *k-mer* set to 64 and a joint analysis of all Svevo libraries, CLC produced 180,108 contigs, with a N50 length of 1121 bp and mean contig length of 883 bp while 57.1% of contigs had lengths ranging between 300 and 700 bp (Additional file 4: Figure S1)*.* The contig number obtained from the analysis of pooled reads was higher than those obtained with any of the four plant combinations of tissues analysed separately, while maintaining a N50 value comparable to that obtained for the best organ-specific assembly, i.e. 1132 bp for pooled ovary and anther organs at anthesis. The Velvet-Oases assembler with *k-mer* size of 71 and 81 produced results similar to the assembly of CLC with *k-mer* 64, with the difference that the joint-analysis of all available reads did not improve the number and mean length of contigs. In particular, with *k-mer* 71 and 81, the analysis of all organs produced 115,798 and 105,743 loci, respectively, with N50 length of 1831 and 2057 bp and mean length of 780 and 1041 bp, respectively. With *k-mer* 64, CLC assembled 159 Mb while Velvet-Oases assembled 90 Mb with *k-mer* 71 and 110 Mb with *k-mer* 81. The assembly performed with CLC with *k-mer* 64 and all four tissues together was selected due to the best overall features including N50, contig length and total size assembled (Additional file 5: Figure S2, Additional file 6: Figure S3).

Gene completeness of the de novo assembly was estimated based on two independent samples of validated wheat genes using a procedure that included counting the number of contigs necessary to reconstruct each gene and then evaluating the corresponding percentage of reconstruction. Increasing *k-mer* size improved the number of reconstructed genes (Additional file 7: Table S4). In CLC, from a *k-mer* size of 41 to 64, the percentage of genes reconstructed increased by 9.6 and 12.7% for chromosome 3B and for the full-length cDNA datasets, respectively.

To assess the complete reconstruction of the two homoeolog genes, a set of 58 *T. aestivum* genes from chromosomes 3A and 3B was compared with the selected assembly (Additional file 8: Table S5, see Methods). In most cases, the percentage of identity between homoeolog genes was between 95 and 100% (Additional file 8: Table S5) while a lower percentage identified paralogous genes or multigene BLASTn families. In total, the de novo assembly allowed for the reconstruction of both homoeologs in 40% of cases, one copy in 12% of cases, less than one copy in 10% of cases, between one and two copies in 29% of cases while the remaining 9% represented genes not reconstructed at all (Additional file 9: Table S6). In some cases, putative paralogs of given genes could also be identified in the assembly but in most cases their sequence identity dropped below 90%.

The comparison with the Benchmarking Universal Single-Copy Orthologs (BUSCO plants dataset), see Methods, composed of 956 gene models, showed a reconstruction of 800 (84%) whole genes with 482 (50%) duplicated while 115 (12%) genes were reconstructed just partially and only 41 (4%) were missing. The comparison of Svevo transcriptome with the publicly available Kronos transcriptome by means of a permissive BLASTn discriminated 13,636 sequences specific to the Svevo cultivar, 3330 of which were functionally annotated as gene families like disease resistance, NADH dehydrogenase, receptor protein kinase, etc. (Additional file 10: Table S7). A manual inspection revealed that in most cases these contigs represented exons, missing in the Kronos assembly.

### Transcriptome annotation

The alignment of assembled contigs versus transcriptomes of nine plant species (Additional File 11: Table S8) resulted in 77,572 (43% of total 180,108) sequences with at least one matching sequence. Out of 77,572 matching transcripts, 22,031 were classified as transcripts with a known function, while 43,969 were classified as putative functional transcripts. Additionally, 11,572 transcripts with low identity were discarded.

Among transcripts with a known function, 14,232 were annotated with BioMart and 6369 transcripts were annotated in BAR+ (see Methods). By unifying both annotations, 15,072 transcripts were finally annotated. Considering the putative functional transcripts, 23,138 transcripts were annotated with BioMart and 10,473 transcripts were annotated in BAR+. Overall, 25,529 transcripts were annotated (Additional file 11: Table S8).

Finally, 154,579 transcripts that could not be annotated either with BioMart or with BAR+ were further used as input for Blast2GO annotation. Of these, 34,728 transcripts (22.5%) were associated to GO (Gene Onthology) terms. In total, 60,257 transcripts were annotated.

Transcripts’ annotation features were summarized based on the annotation results (Additional file 12: Table S4). Considering the 15,072 transcripts with high-confidence annotation (> 90% protein coverage and > 50% identity), 11,144 showed at least one annotation with GO terms and 13,356 showed at least one annotation with Pfam ID. Considering less stringent annotation parameters (> 50% protein coverage and > 30% identity), out of 25,529 transcripts that showed at least one hit with the protein databases, 17,679 were annotated based on at least one GO term and 21,613 based on Pfam ID.

The 15,072 high-confidence annotation transcripts matched 12,347 proteins, while when considering the 25,529 transcripts at the less stringent annotation parameters, 18,437 proteins matching the Svevo transcripts were identified. The summary of these statistics is presented in Additional file 12: Table S4. More in detail, the number of transcripts matching the nine plant transcriptomes are reported in (Additional file 13: Table S9) with the highest number of matches found in *T. aestivum*, *Hordeum vulgare* and *T. urartu* (39.1, 20.9 and 14.5%, respectively). Analyses of transcripts divided by molecular function, biological process and cellular component are presented in Additional file 14: table S10 and Additional file 15: Figure S5.

### SNP identification and validation

**Fig. 1:**
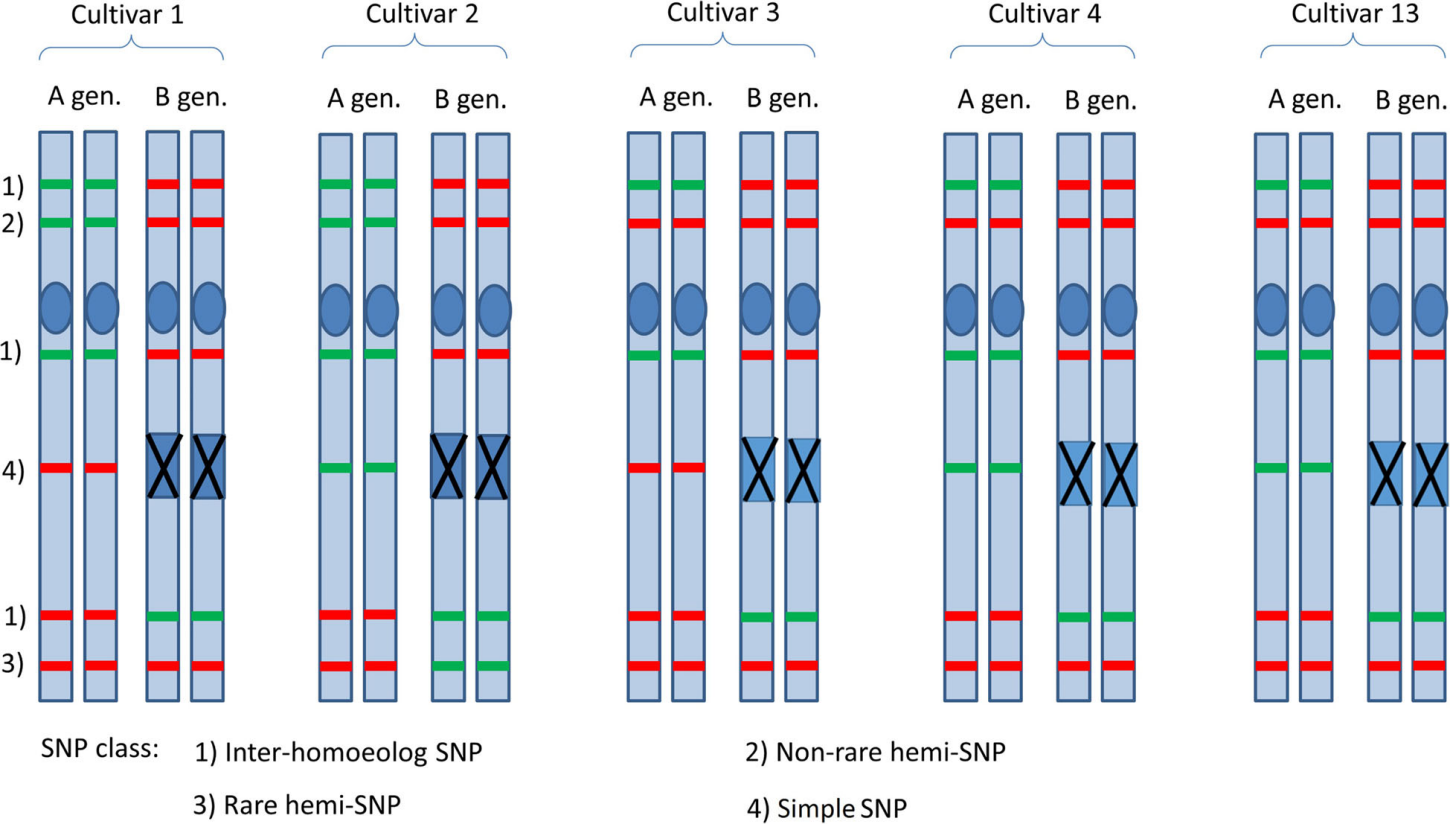
Schematic representation of inter-homoeolog SNPs, hemi-SNPs and simple SNPs for multiple cultivars in allo-tetraploid durum wheat. Inter-homoeolog SNPs represent constitutive homeolog-specific variation. Hemi-SNPs represent homoeolog variation which occurs only on some samples. Simple SNPs represent effective diploid status, in this case following a deletion and thus having on sub-genome as the only allelic complement

**Fig. 2:**
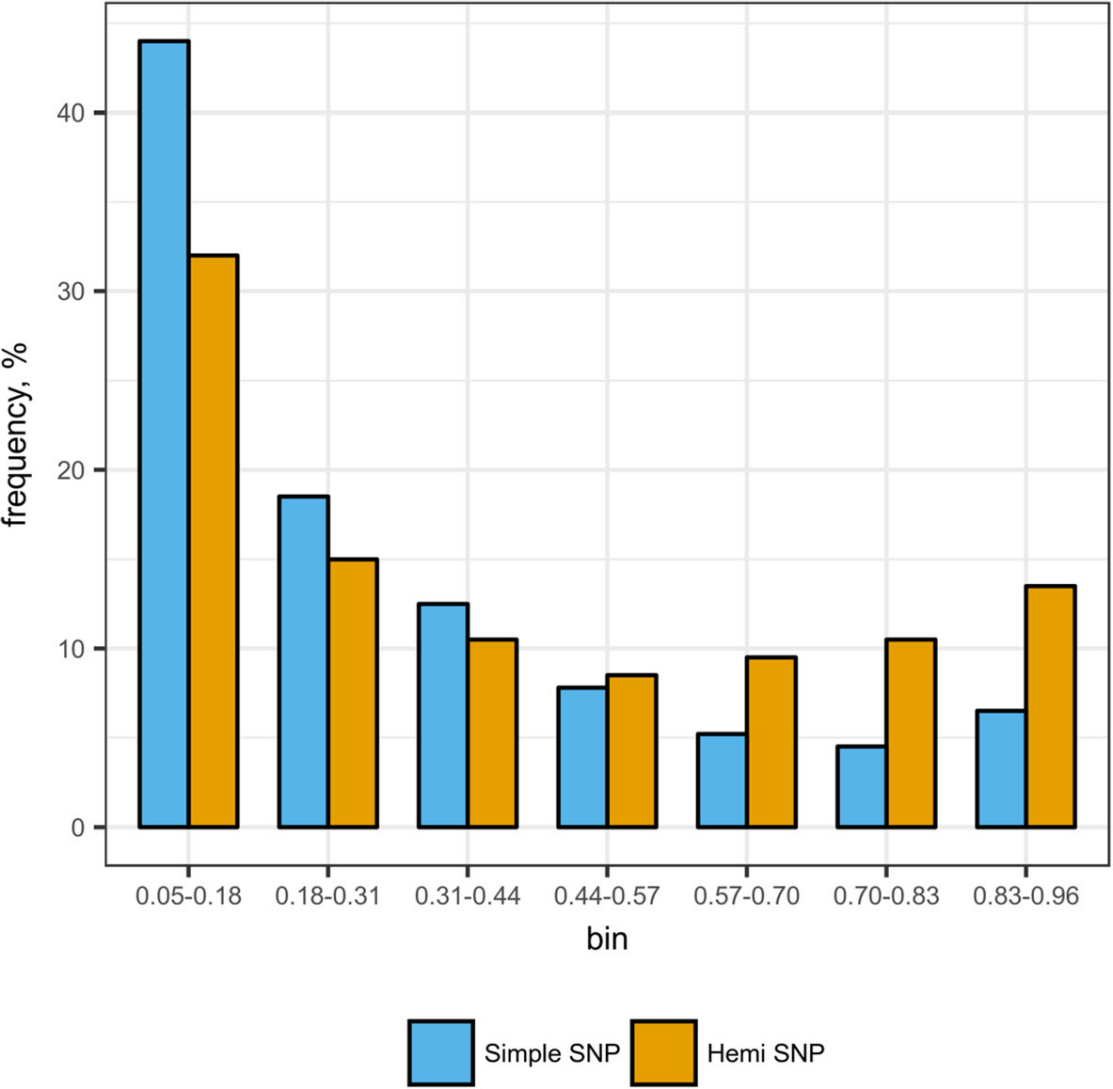
Simple and Hemi-SNP frequency distribution within the 13 varieties. SNP frequency among the 13 cultivars studied. A SNP was considered when at least seven out of the 13 varieties were sufficiently covered. Simple and Hemi-SNP frequencies are presented divided into seven bins. For example, for a given position having three varieties calling non-reference nucleotides, nine with only reference nucleotides and one without enough coverage, it will fall in the bin 0.18–0.31 as it corresponds to a position with 25% of SNPs (3 out of 12)

Across all 13 varieties, a total of 66.6 Gb, 86.4 Gb and 74 Gb paired (2 × 100 bp) reads were sequenced for leaf, root and grain, respectively (Additional file 1: Table S1). The SNP identification procedure produced 747,164 SNPs (Table 1), 9400 of which were considered as biologically unlikely (assembly errors) and were thus discarded given that all varieties, including Svevo itself, were consistently different from the reference. An additional 110,876 SNPs were clearly assigned to the inter-homoeolog, non-varietal hemi-SNP category (Fig. 1, inspired by [15, 16]). Out of the remaining 626,888 SNPs that were classified as varietal-SNPs, 33,747 were single dose, locus-specific SNPs (5.38% of the varietal SNP, with Mendelian diploid behaviour) and 497,783 were considered as varietal-hemi-SNPs, 95,358 of which were non-rare SNPs (i.e. allelic presence in two or more genotypes out of 13 investigated). Hemi-SNP and simple SNP frequency distributions among the 13 cultivars are represented in Fig. 2. In both cases rare SNPs were among the mostly represented classes. Simple SNPs were 11.6% enriched in rare SNP frequency as compared to hemi-SNPs (43.8% vs 32.2%, respectively).

**Table 1:** SNP identification and selection in the 13 durum wheat cultivars

	Number	Percent
a. SNPs		
Simple SNPs	33,747	4.52%
Hemi-SNPs	497,783	66.62%
Inter-homoeolog SNPs	110,876	14.84%
SNPs with more than two genotypes	95,358	12.76%
Assembly discordant SNPs	9400	1.26%
Total SNPs computed	747,164	
b. Selected SNPs		
Hemi-SNPs	4461	56.18%
Simple SNPs	3479	43.83%
Selected SNPs for the wheat chip	7940	

Panel (a) reports the overall SNPs computed, divided in different categories. Panel (b) reports the SNPs selected for the wheat chip with classification between simple and hemi-SNP

**Fig. 3:**
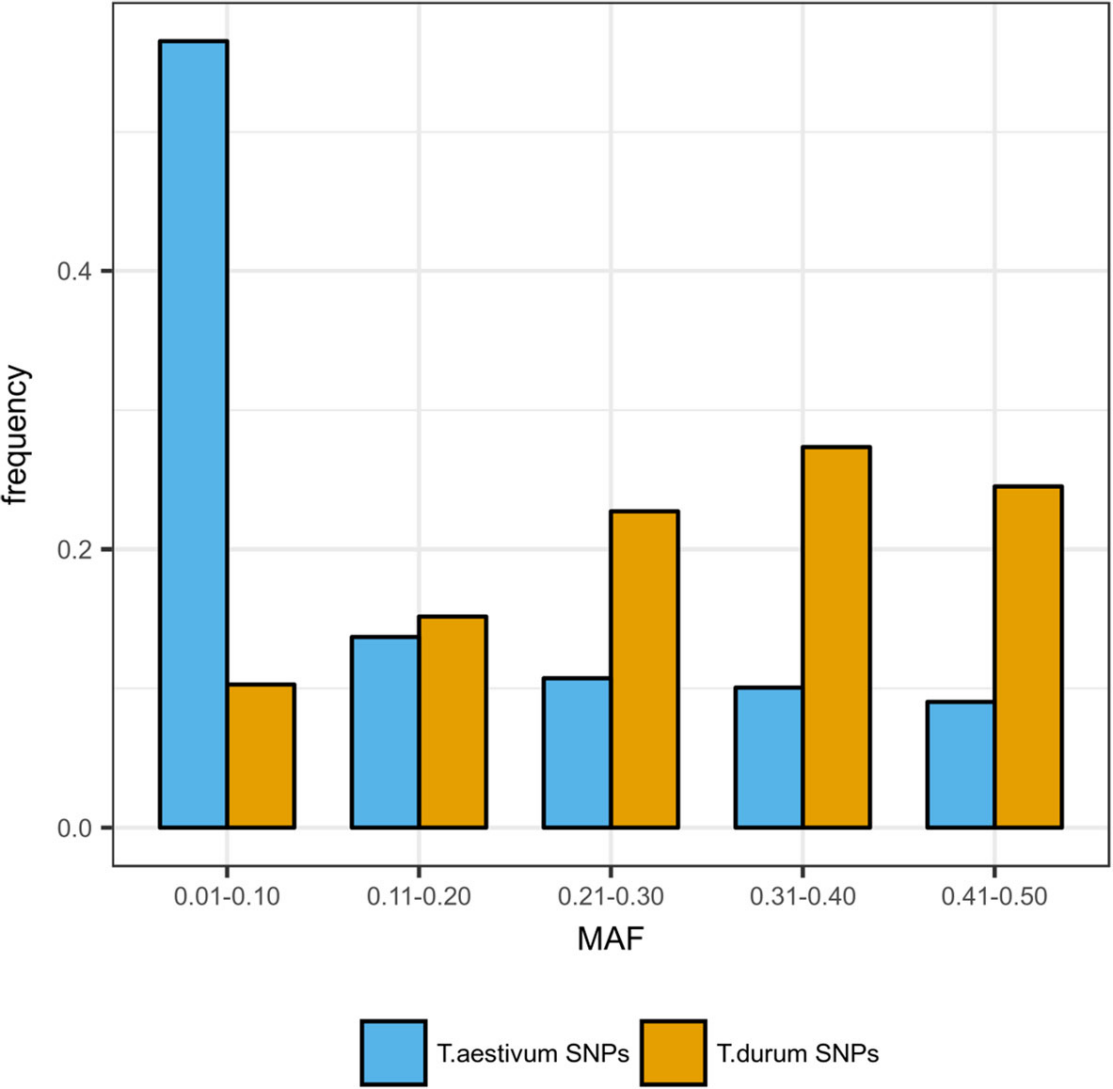
Minor Allele Frequency distribution for *T. durum* SNPs and *T. aestivum* SNPs assessed on the durum wheat panel. MAF comparison of *T. durum-*derived and *T. aestivum*-derived SNPs within a panel of 288 durum wheat accessions as representative of the worldwide breeding germplasm. Distribution of MAF is presented divided into 5 bins

Based on this refined dataset, the 7940 SNPs with the highest Illumina Assay Design Tool (ADT) score were included in the wheat 90 K Illumina iSelect SNP array along with 73,647 *T. aestivum* SNPs and used to assay a worldwide panel of 288 durum wheat elite accessions (Table 2) [17]. Polymorphic SNPs from durum wheat panel were 3738 out of 7237 non-failed assays (51.7%), while for those mined in hexaploid wheat 20,807 SNPs were polymorphic out of 68,865 non-failed (30.2%). As expected, in this *T. durum* germplasm panel the yield of polymorphic SNP sites was higher for the *T. durum* SNP catalogue sites as compared to the *T. aestivum* ones*.* Also, when comparing the *T. durum* to *T. aestivum* SNP groups, the number of failed SNPs was not statistically different (6.5 and 8.8%, respectively, t-test *P* = 0.235). The allelic frequency of the polymorphic SNPs obtained from *T. durum* and *T. aestivum* were compared by analyzing the distribution of Minor Allele Frequency (MAF) (Fig. 3). The MAF distribution of polymorphic SNPs confirms that *T. durum* SNPs exhibited a higher polymorphism level than those obtained from *T. aestivum*, when assayed on a *T. durum* germplasm. However, the *T. aestivum* SNPs also provided a valuable level of informativeness when used on the *T. durum* germplasm as well.

**Table 2:** Number of failed, polymorphic and total SNPs in the assay

	SNPs in assay	Failed SNPs	Polymorphic SNPs
*T. aestivum* SNP	73,647	4782 (6.5%)	20,807 (30.2%)
*T. durum* SNP	7940	703 (8.8%)	3738 (51.7%)
Total	81,587	5485 (6.7%)	24,545 (32.3%)

Informativeness of *T. aestivum* and *T. durum* SNPs included in the iSelect Illumina wheat 90 K SNP array as evaluated on a panel of 288 worldwide durum wheat elite germplasm accessions. Percentage of polymorphic SNPs is based on the number of successful SNPs

### Homoeolog-specific expression in durum wheat

Mapping efficiency of Illumina reads against the *T. urartu* reference gene set ranged between 40 and 70% depending on samples. Out of the 34,879 transcripts in the reference set, 7040 successfully generated at least one phased block of sequences. We generated a total of 11,465 phased blocks and after filtering low-quality blocks, 8189 were retained to produce putative genome-specific transcript sequences. Transcripts edited by means of a single block of phase were 5136, while the remainder were subjected to one or more splits between neighbouring phase blocks. Overall, 75,170 phased SNP sites were used to generate homoeolog-specific sequences, while 38,446 fixed variant sites were edited in both sets to improve mapping efficiency.

**Fig. 4:**
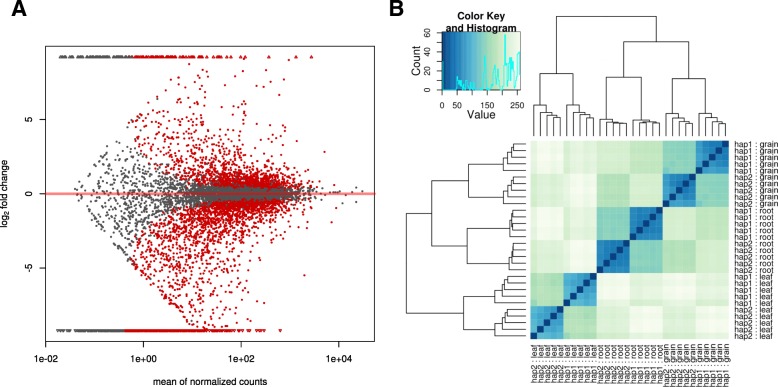
Homoeolog-specific expression. **a** MA-plot of significant differentially expressed homoeologs using GLM fit method. **b** Sample-to-sample heat map of homoeolog-specific expression similarity. Most of variance is accounted between samples, while a lower fraction is contributed by homoeolog-specific expression (hap1 and hap2). As expected, the five pseudo-replicates showed very high similarity compared to that of homoeologs and tissues (grain, leaf, root) distances

**Fig. 5:**
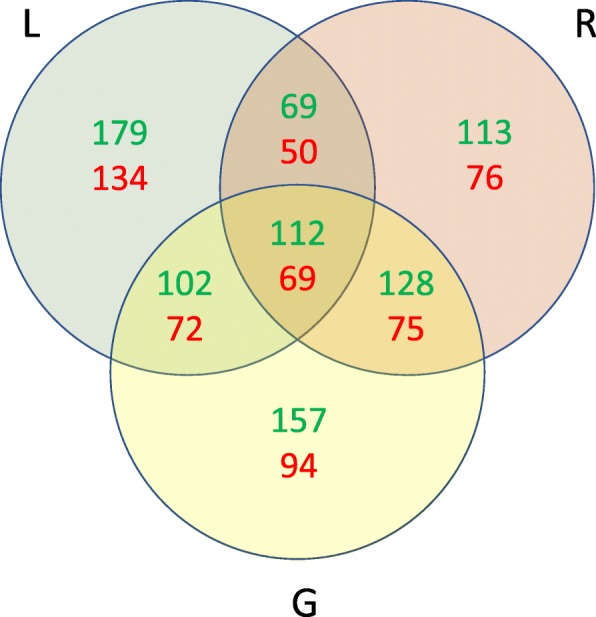
Differentially expressed homoeologs within different tissues. Number of differentially expressed homoeologs as identified with negative binomial test, Bonferroni-corrected *p*-value < 0.01. Tests have been carried between each haplotype pair for a given tissue, (G = grain; L = leaf; R = root). In green is reported genes with over-expression of the A genome, in red those that showed higher expression in the B genome

Among the 7040 genes considered for the analysis, 1808 were found to show a significant differential expression between homoeologs using a multi-factorial GLM fit approach (see Methods) which aimed to find any significant bias in favour of a particular sub-genome by modelling tissue-specific variance. A cluster analysis of expression levels confirmed that most of the variability is accounted by the tissue type, then by homoeologs. Conversely, heterogeneity among pseudo-replicates was limited (Fig. 4). To assess the reliability of our phase reconstruction we investigated whether genes with two or more phased blocks showed non-discordant fold-change direction across blocks. Out of 87 genes considered, 76 showed concordant values, indicating that our de novo homoeologs reconstruction pipeline was able to correctly assign ancestor genome haplotype in most cases (Additional file 16: Figure S6). Moreover, differential expression of splicing variants may also be a cause for incongruent pattern among blocks in some genes. Overall, 1113 genes (15.81%) showed higher expression of the A-genome homoeologs, while 695 genes (9.87%) a significant overexpression of the B-genome. Further, 802, 706 and 820 homoeologs were found to be differentially expressed in leaves, roots and grains, respectively, when adopting tissue-specific binomial test analysis. By grouping genes based on tissues that showed a differential expression of homoeologs, either by a single tissue or by a multiplicity of them (Fig. 5), we observed a constant trend in favour of the A-genome homoeolog; we cannot rule out entirely that this may be caused by alignment bias on the *T. urartu* reference transcripts. However, a higher level of divergence was observed between A and B genome-specific expression in grain tissue than in roots and leaves (*P* = 0.06, t-test). We further investigated the presence of divergent homoeolog expression between tissues to assess the extent of regulatory subfunctionalisation following polyploidisation. We found only 16 genes (< 1%) with contrasting homoeolog-fold-changes across tissues, where none of them were differentially expressed for each of the three tissues. This suggests that homoeolog-specific expression does not mainly correlate with tissue-specific expression (Additional file 16: Figure S6).

**Fig. 6:**
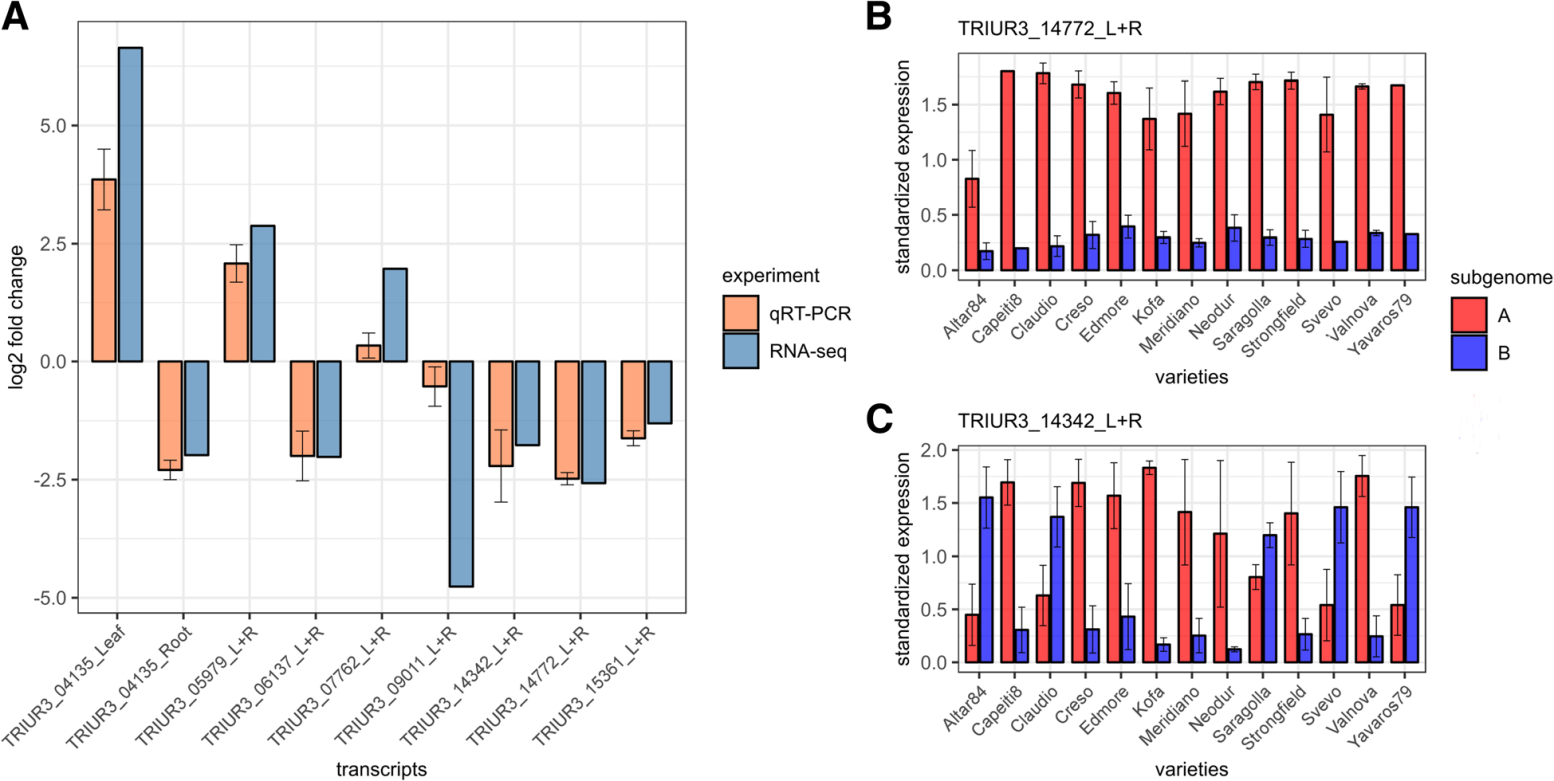
qRT-PCR results summary. **a** Comparison of A/B-homoeologs Log2FC of expression data obtained respectively from RNA-Seq and qRT-PCR validation assays; qRT-PCR data reports values from eight control transcripts assayed on 13 varieties, each in triplicate. The suffix L on the gene ID stands for leaf, while R suffix stands for root. **b** Homoeolog-specific qRT-PCR standardized expression data across 13 varieties for transcript TRIUR3_14772 with a constitutive up-regulated A-genome. C) Homoeolog-specific qRT-PCR standardized expression data across 13 varieties for transcript TRIUR3_14342 which reported a variety-specific sub-genome dominance. The whiskers are lower and upper standard errors

Using the criteria described in Methods, we developed qRT-PCR assays for both A and B subgenomes for eight transcripts illustrated in Additional file 17: Table S11 (32 assays, 16 for leaves, 16 for roots, for each of the 13 varieties). We confirmed one transcript (TRIUR3_04135) with alternate expression between leaf and root tissues with the B-genome homoeolog up-expressed in leaves and the A genome homoeolog up-expressed in roots. Four transcripts (TRIUR3_06137, TRIUR3_09011, TRIUR3_14772 and TRIUR3_15361), as indicated by RNA-Seq data, reported A-genome homoeologs up-expressed in both roots and leaves while two transcripts (TRIUR3_05979, TRIUR3_07762) confirmed the opposite pattern (B-genome homoeologs up-expressed in both tissues). All the above-described results for seven transcripts were consistent with those obtained from RNA-seq experiment. Moreover, for all the seven transcripts the expression was consistent among all the varieties and tissues (Additional file 18: Table S12 show expression of all genes across 13 varieties). The average expression for the 13 varieties for these seven transcripts and their standard errors are reported in Fig. 6. One transcript (TRIUR3_14342) showed the A-genome homoeologs up-expressed only in eight varieties and B-genome homoeologs up-expressed in five varieties (Additional file 18: Table S12).

## Discussion

### Selection of reference assembly

Our study adds novel transcriptome information to the available *T. turgidum* ssp. *durum* reference from cv. Kronos [8]. We report a de novo transcriptome assembly for Svevo, a modern Italian durum wheat reference for semolina quality, grain yield and adaptation to Mediterranean environments [18]. Based on a previous survey of diversity in a worldwide panel of elite durum wheat [19] the two varieties showed a genetic similarity (identity by state similarity, IBS) equal to 0.17, with an average genetic similarity across all the cultivars equal to 0.29. Thus, the genetic relationship between Svevo and Kronos can be considered lower-than-average when considering the modern durum wheat germplasm. The sampled tissues and NGS methodology of the herein reported Svevo transcriptome were similar to those used for Kronos. Young leaves (coleoptiles), roots at the seedling stage and developing grains at 20 days post-anthesis were common to both cultivars, with the exception that whole spikes (at booting stage) were collected for Kronos while ovaries and anthers (sexual organs only) were collected for Svevo.

The sequencing data generated in the two experiments were similar as well, approximating the 400 million reads for Svevo and the 500 million reads for Kronos; however, the Illumina reads generated for Svevo were up to 150 bp long while Kronos reads were up to 100 bp long. The two assembled transcriptomes were similar in total size (159 Mb for Svevo and 181 Mb for Kronos). The Svevo assembly, compared to Kronos, included a higher contig number (180,108 vs. 140,118) with a lower mean contig length (883 vs. 1299 bp). This apparently negative result is due to the more stringent assembly parameters used herein, which allowed for a reduced redundancy of the assembly and for the identification of exons and whole genes missing in the Kronos assembly.

For the Svevo assembly, different strategies and multiple parameters were tested with the aim of producing a final assembly as complete as possible. Basically, two strategies were pursued, one based on CLC-Genomics Workbench and one pairing the tools Velvet and Oases. One of the most commonly used tools, Trinity [20], was tested as well; however, after a few tests it was abandoned since the results obtained did not significantly improve the quality of the assembly, coupled with an impractical request of resources. The *k-mer* length is a critical parameter in assembling a transcriptome using De Bruijn graphs, a data structure exploited in most of NGS de novo assemblers. In practice, when a contiguous assembly is the primary goal and the loss of lowly expressed transcripts is not a main concern, a large *k-mer* length is preferred. On the other hand, small *k-mer* length is often used to capture poorly expressed transcripts, resulting in more fragmented and diverse transcripts [21, 22]. Krasileva et al. [8] used a mixed approach based on merging the results from multiple *k-mer* assemblies. Conversely, empirical data from our assemblies showed that CLC-derived assemblies with a shorter *k-mer* turned out to be a subset of assemblies with longer *k*-mer. In the case of Svevo, 45 different assemblies were tested in total. Within the scope of the current study, the ones with the largest *k-mer* values produced the best assemblies with the mostly contiguous sequences. In particular, within a set of 16,803 *T. aestivum* full-length cDNA sequences [11, 23] the best assembly reconstructed entirely 79.9% of genes. Considering the taxonomical and genetic distance between the two species, we can consider that the percentage of genes completely reconstructed is very likely to be even higher. Coverage of at least one homeolog was assessed to 84% but the reconstruction of both copies of homoeolog genes of the tetraploid durum was more difficult, with an estimation of 40% of sequences based on a benchmark of 58 genes from whole genome assembly.

With current technologies, a de novo transcriptome of a diploid eukaryotic species encompasses hundreds of thousands of genes, including multiple isoforms and very low expressed genes. The situation is even more complicated in transcriptomes of polyploid organisms such as *Brassica* ssp. [24–26]. In hexaploid wheat, a total of 133,090 high-confidence genes have been annotated based on the genomic sequence of flow-sorted single-chromosome arms [12, 27]. In hexaploid wheat, the use of the emerging single-molecule real-time (SMRT) sequencing technology (Pacific Biosciences) allowed for a massive sequencing of full-length non-chimeric reads, 74.6% of which corresponded to a complete open reading frame [28]. This technology allowed for a detailed investigation of the transcriptome of developing grains and led to obtain a more complete picture of the gluten gene transcript, including the identification of many pseudogene transcripts and a clear discrimination between homoeologs and paralogs. However, the inherently higher cost and sequencing error rate has so far prevented a more widespread use of this technology.

### Functional annotation of *T. durum* transcriptome

The use of multiple complementary approaches for transcriptome annotation allowed us to functionally annotate a sizeable number of sequences (78%) using the nine transcriptomes of closely related plant species. This percentage is similar to the annotation results reported for the Kronos transcripts [8]. As expected, annotated transcripts showed high similarities with *Triticum aestivum, Hordeum vulgare* and *Triticum urartu* suggesting similar functions. Transcripts that were not annotated could be durum-specific transcripts, unannotated genes, non-coding transcript sequences or regions that underwent substantial mutations. One of the reasons for the relatively limited number of transcripts assigned with high confidence to a very specific function is a low coverage of the matched protein by the transcript. Only transcripts that covered the matching protein were extracted and further analysed for functional annotation. Another factor that could have influenced the annotation efficiency is the fluctuation in expression level and the insufficient coverage achieved for a portion of transcripts. Nevertheless, the transcripts annotated in this work provide valuable information towards the identification of the expressed portion of the genome in *Triticum*. Additionally, this study contributes towards the production of a more representative and well-annotated database of expressed genes in durum wheat.

### Transferability of the SNP panel

To our best knowledge, this is the first study providing a high-quality and very extensive dataset of *T. durum*-specific SNPs. The transcriptome sequencing approach can be considered as an open platform for high-throughput SNP identification, as currently adopted for many plant species of agronomic interest [29–32]. Moreover, SNPs were identified among a set of hallmark cultivars representing a wide range of genetic diversity and widely diffused in the Mediterranean region and worldwide. Therefore, these SNPs are likely to be highly informative and particularly suited for genetic mapping (e.g. GWAS) and breeding applications such as marker-assisted selection in the cultivated durum wheat germplasm.

Within the 13 sequenced varieties, rare SNPs were the most represented, for both hemi-SNPs and simple SNPs (Fig. 2). Interestingly, the frequency of the rarest simple SNPs is 11.6% higher than the frequency of the corresponding class in the hemi-SNPs. This may reflect multiple causes including, among others, the stringent definition given in this paper to simple SNPs, namely either the presence of both homoeolog sequences in the de novo reference with assignment of reads only to the correct chromosome or deletion of one of the homoeolog chromosomes with a consequent lack of reads from the other chromosome.

We assayed the SNP information content by including a subset of 7940 tetraploid SNPs to the iSelect Illumina 90 K wheat assay [17] where also a considerable portion of the functional *T. aestivum* SNPs were still informative in the *T. durum* germplasm (28% with MAF > 0.2), making the iSelect Illumina 90 K assay a valuable option for genotyping also tetraploid wheat, as confirmed by several mapping studies [33–35]. Since *T. aestivum* derived from *T. turgidum* ssp. *dicoccum* or *T. turgidum* ssp. *durum* only 8–10,000 years ago, *T. aestivum* SNPs are expected to retain an appreciable level of polymorphism present in *T. durum,* a feature reinforced by the evidence of gene flow which occurred between tetraploid and hexaploid germplasm [36]. On the other side, the relatively high peak of SNP frequency in the lower MAF class for the *T. aestivum* set of SNPs was also expected, considering that after polyploidisation the germplasm pools of the two species, and particularly the elite germplasm cultivated nowadays, have undergone multiple events of population size reduction, drift and selection, and novel introduction [4, 37]. Moreover, the herein reported novel *T. durum* SNP dataset was biased towards high MAF values because of the SNP selection process that was based on SNPs that were confirmed in at least three of the 13 reference cultivars. The novel *T. durum* SNPs dataset is also valuable in future work aimed at the design of additional dedicated SNP arrays. However, the application of this tool in genetic studies (diversity analysis, genetic mapping, etc.) involving wild or ancient tetraploid wheats should be carried out with caution, given that SNPs were identified among elite durum wheat cultivars.

### Survey on homoeolog-specific expression

We investigated the extent of homoeolog-specific expression in *T. durum*, a species putatively resulting from the hybridisation event between *T. urartu* (A-genome, 2n = 2x = 14) and *Aegilops speltoides* (B-genome, 2n = 2x = 14), which generated an allotetraploid chromosomal set (2n = 4x = 28). A draft genome assembly of *T. urartu* has been published [38], and while writing of this manuscript an improved version has been released [39]. Also, a reference genome for the D progenitor *Aegilops tauschii* has been released [40]. However, the absence of a B-genome draft assembly is still hampering the possibility to classify homoeolog-specific nucleotide variants in an unbiased way. A simple alignment of sequence data from *T. durum* to the A-genome representing *T. urartu* to detect B-genome specific variants would erroneously classify any intra-genome genetic variant occurring between the ancestral *T. urartu* genome and the domesticated A-genome. Moreover, short NGS reads (100 bp) are known to suffer alignment bias in favour of the haplotype represented in the reference sequence [41], which prevents correct identification of allelic/homoeolog-specific expression.

To circumvent these issues, we adopted a method based on the de novo reconstruction of haplotypes from Illumina paired-end reads (2 × 100 bp) coupled with a double-reference mapping procedure. Mapping RNA-seq reads on the double reference allowed us to distinguish between (i) reads mapping to one of the two alternative transcripts (i.e. those containing one or more homoeolog-specific variants) and (ii) ambiguously mapped reads for which no homoeolog-specific variant is present. The former ones were then used to estimate differential expression levels among the two putative homoeologs.

Although previously described precautions were taken, still 15.81% of genes showed higher expression of the A-genome homoeolog versus 9.87% of the B-genome homoeolog (Fig. 5). If, on one side, this indicates that about as many as 10% of the genes are more expressed in the A-genome, on the other side, it may indicate a possible subfunctionalisation of a high number of genes. Although the mechanisms of subfunctionalisation is still poorly understood it appears that the A-genome was favoured in this process, thus confirming recent observations in hexaploid wheat [42]. Our results have also been validated by qRT-PCR where seven genes out of eight reported the same homoeolog-specificity pattern for all varieties as indicated by RNA-Seq data and one gene showed a variety-specific over-expression of either A-genome or B-genome, suggesting that homoeolog-specific expression contributes to the diversity within the elite germplasm. Indeed, TRIUR3_14342 showed the homoeologs expression pattern across varieties related to the ancestry relationship [19]. Altar84, Claudio, Saragolla, Svevo and Yavaros79, belonging to CIMMYT germplam, reported up-regulated B-homoeolog, while other varieties related to Italian and North American germplasm reported up-regulated A-homoeolog.

## Conclusions

This study presents a de novo transcriptome assembly of Svevo, a modern elite durum wheat cultivar widely used in breeding programs. Among several de novo assembly methods, we selected the CLC Workbench assembly and showed that the maximum value, *k* = 64, for *k-mer* parameter generated contig length close to the expected length distribution of transcripts and genes. This transcriptome assembly expands the existing publicly available reference transcriptome of the durum wheat variety Kronos and contributes towards a novel and more complete transcriptome information to the ongoing genomics studies in tetraploid durum wheat. The transcriptome reported an in silico level of completeness of 84% with 78% of the reconstructed sequences being functionally annotated, including GO terms and PFAM domains. The RNA-seq data of the 13 elite durum wheat varieties provides a relevant number of novel *T. durum*-specific SNPs, a valuable resource to more effectively characterise wheat QTLs (Quantitative Trait Loci) and implement genomics-assisted breeding programs in the cultivated durum wheat germplasm. Using a double-reference mapping procedure, we first investigated the homoeolog-specific expression in durum wheat and validated the method. This latter exercise suggested that homoeolog-specific expression may contribute to the diversity across varieties and further large-scale studies may provide a better understanding of the interplay of two subgenomes across germplasm.

## Methods

### Plant material

The Italian durum wheat (*Triticum turgidum subsp. durum* Desf.) cv. Svevo, released in 1996 (CIMMYT line/Zenit) was selected for the reconstruction of the durum wheat reference transcriptome, as it has been a quality and productivity reference variety of durum wheat in Italy for more than a decade. A selection of 13 varieties (including Svevo) was used to produce RNA-Seq data in order to mine for functional SNPs given their relevance as breeding material (Additional file 1: Table S1). Plants were grown in growth chamber under optimal conditions for wheat (long days 16/8 h day/night photoperiod regime at 20/16 °C day/night temperature regime).

### RNA extraction, library preparation and sequencing

To produce a collection of transcripts as complete as possible for Svevo cultivar, four different combinations of tissues were selected: (i) coleoptile and leaves at the seedling stage, (ii) apex of seminal roots at the seedling stage, (iii) ovaries and anthers at beginning of anthesis (Zadoks 60) and (iv) developing grains at the growth stage Z70 [43]. Total RNA was isolated from 100 mg of each tissue using Total Spectrum Plant RNA (Sigma-Aldrich) according to the manufacturer’s instructions. RNAs were quality checked using the RNA 6000 Nano kit on a 2100 Bioanalyzer (Agilent) and accepted when RIN ≥ 8 and quantified on Nanodrop spectrophotometer (Thermo Scientific). For each tissue derived from Svevo, two cDNA libraries (eight in total) were constructed from 4 μg total RNA using the Illumina TruSeq RNA Sample preparation kit according to the manufacturer’s protocol. After PCR enrichment, cDNA fragments were separated on a 2% low agarose 1X TAE gel; two size fractions, one with an average fragment size of 280 bp and another with a fragment size ranging from 380 to 480 bp were extracted and then purified using the Gel-Extraction kit (Qiagen). The two libraries from each tissue were pooled together and quantified with the Bioanalyzer using the High Sensitivity kit (Agilent). The libraries were loaded as a pool on a Cluster Generation machine in a single Illumina flowcell lane following the standard Illumina protocol. A paired-end sequencing protocol was conducted with the Illumina GAIIx generating 100 bp and 150 bp reads in pairs, with different proportions depending on the sample (Additional file 2: Table S2).

Thirteen elite varieties representing the diversity of the worldwide durum germplasm (Additional file 1: Table S1), including the selected reference cv. Svevo, were chosen to produce cDNA libraries from leaf, root and grain (cDNA libraries of Svevo were repeated for this scope to avoid batch effect in the expression analyses).

RNA isolation, library preparation and sequencing were carried out as described above. The libraries for the 13 durum wheat varieties were prepared as previously described for Svevo, with gel size selection of only 500–600 bp fragments. The purified libraries were used to produce two pools, 6-plex and 7-plex, and after quantification on the Bioanalyzer, were loaded on two lanes of a HiSeq2000 sequencer run. 100 bp paired reads were produced with the standard Illumina pipeline.

### Svevo de novo transcriptome assembly and validation

rNA [44] was run with default parameters (except for “min-size” 50) to trim low-quality regions and to remove possible contaminants (*E. coli*, mitochondrion and chloroplast). Trimmed paired reads from either single tissues or from the bulk of the four tissues were initially used to create de novo transcriptome assemblies using either CLC-Genomics Workbench v5.1 (CLC Bio, Aarhus, Denmark) or Velvet [45] paired with Oases [46]. A total of 20 and 25 assemblies have been produced with CLC and Velvet, respectively, at different *k-mer* sizes (CLC: 41, 51, 61 and 64, the maximum allowed by the software; Velvet: 41, 51, 61, 71 and 81). For CLC, bubble size was set to 50. The minimum contig length was set to 300 bp and paired reads were scaffolded (Additional file 4: Figure S1, Additional file 19 Figure S7-S16, Additional file 3: Table S3).

The completeness of the assemblies was assessed in terms of percentage of gene reconstruction. De novo contigs were initially aligned against (i) a set of CDS from 197 genes from *T. aestivum* chromosome 3B-reference sequences, derived from 10 selected assembled genomic contigs (GenBank accessions FN564426.1, FN645450.1, FN564427.1, FN564428.1, FN564429.1, FN564430.1, FN564431.1, FN564432.1, FN564433.1, FN564434.1 from [3]) and (ii) a set of 16,803 *T. aestivum* full-length cDNAs [11, 23] using BLASTn with e-value 1E-50 and minimum identity > 80%. To parse the results, an internally developed Perl script was used to take into consideration a blast hit if any of these conditions were satisfied: 1) when hit length ≥ 60% contig length; 2) when hit length > 80% gene length; 3) when hit length ≥ 200 bp. Moreover, for each gene the script returned the number of contigs necessary to reconstruct it and the percentage of gene span reconstructed, i.e. bases covered by at least one hit.

To assess whether both homoeolog copies of each gene were successfully reconstructed in the de novo assembly, a set of 58 pairs of chromosomes 3A- and 3B- homoeolog genes of *T. aestivum* deposited at NCBI database (http://www.ncbi.nlm.nih.gov) (Additional file 7: Table S4) were used. Homoeolog genes were initially compared to estimate the level of identity of each pair (Additional file 8: Table S5). Then, the 58 genes from chr. 3A were compared against the de novo contigs using BLASTn with e-value 1E-35 and minimum identity 80%. For each gene, the cumulative length of the matching hits divided by the gene length was computed and defined “gene coverage” (Additional file 9: Table S6, Additional file 10: Table S7). Hits with an identity close to 100% were considered as deriving from the A genome and hits deviating at most 1% from the paired identity as deriving from the B genome. Overlapping hits were not included in the “gene coverage” count, independently of their identity percentage.

A more general assessment was the comparison of the transcriptome with the plants dataset of BUSCO [47]. BUSCO provides quantitative measures of the completeness of genomes and transcriptomes in terms of expected gene content. Genes that make up the BUSCO sets for each major lineage were selected from orthologous groups with genes present as single-copy orthologs in at least 90% of the species. BUSCO was run with parameters -l plantae -m trans -c 8.

The Svevo transcriptome assembly was compared with the publicly available Kronos transcriptome assembly by means of a BLASTn with a permissive e-value of 1E-05.

### Transcriptome annotation

Annotation of the reconstructed transcriptome was based on a comparative genomics approach. The approach relies on the BLASTx alignment with maximum e-value 1E-03 between transcripts and proteomes of nine different plant species that have already been annotated (Additional file 11: Table S8). The proteome sequences were downloaded from Ensembl Plants Database in FASTA format (Release 18). For the annotation process two parameters were considered crucial: the percentage of identity and the transcript coverage with respect to a matching protein [48]. Transcripts with protein coverage > 90% and identity > 50% were classified as with a known function, while transcripts with protein coverage > 50% and identity ≥30% were classified as with a putative function. The latter classification includes the first. Both sets were annotated with BioMart [49] and BAR+ [50, 51], both used with default parameters. BioMart is a highly customizable data mining tool of Ensembl while BAR+ is a server for protein functional annotation (Gene Ontology), structure and ligands annotation (Protein Data Bank), structural classification of proteins (SCOP), protein domains annotation (Pfam) and allows to compute a 3D model providing an alignment based on Cluster HMM (HMM profile).

Additionally, Blast2GO v.3.0 [52] was used for functional annotation of transcripts that were not annotated either with BioMart or with BAR+. Within Blast2GO, transcripts were compared with BLASTx with e-value 1E-03 versus the NCBI *Viridiplantae* plant database. GO terms associated to BLASTx hits were retrieved based on the GO Mapping process.

### Identification of SNPs within the transcriptome

The quality-trimmed and contaminant-free reads of the 13 varieties were independently aligned versus the selected Svevo de novo transcriptome (minimum similarity 0.8, minimum aligned length 0.9) and SNPs called with CLC-Genomics Workbench v5.1 (window length 11, maximum gap and mismatch count 6, minimum central quality 20, minimum average quality 15, minimum coverage 8, minimum variant frequency 10%, sufficient variant count threshold 1000, required variant count threshold 4).

SNP calls in each single variety were combined with an internally developed Perl script computing a table of SNPs for the 13 varieties. Only positions in the reference with sufficient information (coverage of at least eight reads) for at least five varieties were considered in the table. These SNPs were also used in Wang et al. [17]. Each row of the table, corresponding to a hypothetical SNP in at least one variety, was then classified, with another internally developed Perl script, as either locus-specific SNP, hemi-SNP, inter-homoeolog SNP, position with multiple genotypes or potential misassembly (Fig. 1, inspired by [15, 16]). A SNP was classified as a potential misassembly when all varieties were homozygous and consistently different from the reference. The remaining SNPs were classified as simple SNPs, or locus-specific SNPs, when only homozygous calls were observed in all varieties, hemi-SNPs when both homozygous and heterozygous calls were observed, inter-homoeolog SNPs when all varieties exhibited only heterozygous calls. Considering that in this work only inbred lines were used, a heterozygous call supposedly corresponds to a difference between two homoeolog chromosomes and not to a difference between the two alleles of the same chromosome. Complex situations with more than two genotypes, e.g. some varieties homozygous for the reference allele, some homozygous for the alternative allele and some others heterozygous, were classified separately.

### SNP validation

Genotypic data from a panel of 288 durum wheat accessions representative of the worldwide breeding germplasm were used for the SNP validation. The durum panel was analysed with the Illumina Infinium 90 K iSelect SNP wheat chip, which carries a total of 81,587 functional SNPs [17], 7940 of which were *T. durum-*specific based on the RNA-seq cultivar characterisation. In the durum wheat panel, the number of total SNPs, failed SNPs and polymorphic SNPs were counted for both hexaploid wheat (*T. aestivum*) and durum wheat. The MAF distribution was inspected for the polymorphic SNPs in both hexaploid and durum wheat.

### Homoeolog-specific expression in durum wheat

A total of 384 million RNA-seq paired reads generated from the previously described eight different libraries (four tissues, two libraries with different size for each tissue) of *T. durum* cultivar Svevo were altogether aligned to *T. urartu* reference transcript sequences [38], using Bowtie2 relaxed parameters (−-very-sensitive-local -N 1) [53]. After re-aligning reads around INDELs (small INsertions-DELetions) with GATK RealignerTargetCreator [54, 55] SNPs and INDELs were called using GATK UnifiedGenotyper (down-sampling coverage at 5000). HAPCUT software [56] was used to perform phase reconstruction of polymorphic sites and infer homoeolog-specific haplotypes. A custom Perl script was then used to generate homoeolog-specific sequences from the alignments to the *T. urartu* original sequences, only considering phases where assembly was accomplished with a Minimum Error Correction (MEC) score of 10 or lower and showing at least 90% of variant sites to be phased as expected based on the *T. urartu* reference haplotype. Reconstructed haplotypes with 90% or greater matching to *T. urartu* reference were labelled as derived from the A-genome, while their complementary counterpart as derived from the B-genome. Transcripts where phase assembly produced two or more discontinuous blocks were split accordingly.

Illumina RNA-seq paired reads from leaf, root and grain tissues for each of the 13 durum varieties were mapped against the new reference set containing both homoeolog-derived sequences using BWA aligner with default parameters [57]. Reads ambiguously mapping to more than one position (i.e. to both alternative transcript sequences) were removed and remaining reads were counted as homoeolog-specific reads. Reads mapping as proper pair were counted only once. Given the relatively low abundance of homoeolog-specific reads in lowly expressed genes, read counts from the 13 varieties were merged to five artificial replicates (Altar84 + Capeiti8 + Claudio; Creso + Edmore + Kofa; Meridiano + Neodur + Saragolla; Strongfield + Svevo; Valnova + Yavaros79).

Differential expression analysis of homoeologs was performed using the DESeq package [58]. Two different approaches were used. A multi-factorial analysis accounting for variance among tissues was performed using a Wald test over the general linear models of (i) tissue-only variance fit and (ii) tissue plus homoeolog variance fit. A second approach relied on a single negative binomial test, for investigating differentially expressed homoeologs within each tissue separately, without considering tissue-specific variance. Tests were considered significant with an adjusted *p*-value equal or below 0.001 for the GLM Wald test and 0.01 for the tissue-specific binomial test; the log2 fold-changes where filtered out for absolute values above 7. Dispersion curve was estimated with a pooled strategy as described in DESeq manual [59].

### qRT-PCR validation of homoeolog specific differentially expressed transcript

In order to confirm the consistency of homoeolog specific expression data obtained from RNA-seq experiment, we aimed to verify through qRT-PCR the expression profile of a subset of differentially expressed transcripts. To do this, for each of the 13 varieties, six seeds were grown in a paper-roll in a growth chamber at controlled light and temperature conditions [60]. Samples of leaf and root tissues were collected after 7 days of growth. RNA was isolated from tissues of three biological replicates each containing tissues from three-five seedlings. Tissues were homogenized in liquid nitrogen and RNA was extracted using the NucleoSpin® RNA Plant extraction kit (MACHEREY-NAGEL, Dűren, Germany) following the manufacturer’s protocol. RNA quality was verified on 1% agarose gel and quantity was determined using Eppendorf BioPhotometer D30. All RNA samples were brought to the same concentration prior to reverse-transcription reaction. Reverse-transcription was performed using the QuantiTect® Reverse Trascription kit (QIAGEN, Hilden, Germany) according to the manufacturer’s instructions. cDNA of all the samples was pooled and three serial dilutions [1:4] were performed. The pooled cDNA dilutions as well as a control template (NTC) were included in each qPCR analysis as a control. The differentially expressed transcripts were chosen based on the abs(logFC) > 2 or > 1 between the homoeolog-specific contigs where A or B genomes are up-expressed in both leaves and roots, as well as on the alternate expression of A and B subgenomes between leaf and root tissues. In order to maximize the chances of the gene-specificity of the assay, we chose for primer design differentially expressed transcripts that had no paralogs or tandem duplications in the reference bread wheat IWGSC Refseq v1.0 [61] and wild emmer wheat genomes [14]. To design the subgenome specific assays we aligned the two homoeolog sequences to each other to identify the homoeolog SNPs; at least one of the primers of each assay was designed to include the 3′-end homoeolog SNPs. We used Primer3 [62, 63] to design the primers using the following specifications: Tm 55–65 °C and CG content of 50–60% and maximum amplicon length was kept < 200 bp (Additional file 17: Table S12). In our study, a gene coding for actin was selected as a housekeeping gene [64, 65]. The qPCR was carried out using the SYBR GREEN and 7500/7500 Fast Real-Time PCR System (Applied Biosystems, Foster City, CA, USA). The PCR conditions were 50 °C for 2 min, 95 °C for 2 min followed by 40 cycles of 95 °C for 15 s and 60 °C for 1 min. The expression of the transcripts was normalized using the housekeeping gene actin. Moreover, to have comparable expression values across the thirteen varieties, we standardized the expression value on the mean expression of two homoeologs in each variety.

## Additional files

Additional file 1: **Table S1.** Durum wheat accessions description and RNA-Seq data. Accession name, pedigree, accession feature and the constitutor for the 13 varieties used for RNA sequencing and SNP discovery are reported along with the associated sequencing yields for each of the three sampled tissues. (XLSX 11 kb)

Additional file 2: **Table S2.** Summary of RNA sequencing for durum wheat cv. Svevo cDNA libraries. Two libraries, named “a” and “b”, were constructed for each tissue. They differ on fragment size recovered by gel-electrophoresis. For each library, tissue, fragment size, number of raw reads, total base pairs and average read length are reported. Average read length is proportionally reflecting the amount of 150 bp sequencing and 100 bp sequencing. (XLSX 10 kb)

Additional file 3: **Table S3.** Detailed report of the de novo assemblies. (XLSX 15 kb)

Additional file 4: **Figure S1.** Assembly contigs size distribution. Contigs size distribution in the selected assembly (CLC with *k-mer* size = 64). (PDF 113 kb)

Additional file 5: **Figure S2.** Evaluation of de novo assemblies. Comparison at different *k-mer* values (i.e. k41, k51, k61, k64) and with different tools among different assemblies. (A) and (B) refer to assemblies computed with CLC and indicate, respectively, number of contigs and N50. (C) and (D) refer to Velvet-Oases assemblies. (PDF 601 kb)

Additional file 6: **Figure S3.** Validation of assemblies. The best assembly was validated versus two datasets: (A) full-length cDNAs [11, 23]; (B) *Triticum aestivum* chromosome 3B genes. Bars represent the percentage of genes reconstructed at least in 80% of their length. Different colors represent the number of different contigs necessary to reconstruct the genes. (PDF 104 kb)

Additional file 7: **Table S4.** List of the 58 wheat genes from chromosome 3 with the relative accession numbers. (XLSX 12 kb)

Additional file 8: **Table S5.** BLASTn results between the homoeolog genes of *T. aestivum* chromosome 3. (XLSX 16 kb)

Additional file 9: **Table S6.** Homoeologs reconstruction. Number of copies reconstructed among the 58 genes (Additional file 6: Table S4) of “Supplementary File 2” in the de novo assembly. (XLSX 9 kb)

Additional file 10: **Table S7.** List of Svevo private contigs with GO annotation derived from BLASTn comparison between Svevo and Kronos contigs. (XLSX 338 kb)

Additional file 11: **Table S8.** Transcriptomes of the plant species used for BLASTx. Plant species from Ensembl Plants used for BLASTx alignment of transcripts, to infer functional annotation. (XLSX 9 kb)

Additional file 12: **Figure S4.** Merged BioMart and BAR+ annotations. Venn diagram of transcript annotations with Ensembl and BAR+. On the left, at > 90% protein coverage and > 50% identity a total of 15,072 transcripts were annotated; while, on the right, at > 50% protein coverage and > 30% identity a total of 25,529 transcripts were annotated. (PDF 399 kb)

Additional file 13: **Table S9.** Annotation Statistics. Annotation statistics for the Svevo RNA-seq transcriptome assembly divided into high and low confidence annotation. Annotations with either BioMart or BAR+. Gene Ontology (GO) computed with Blast2GO (B2GO). Three bottom lines from Blast2GO reports. (XLSX 10 kb)

Additional file 14: **Table S10.** Unique proteins matching with *T. durum* transcripts. Unique proteins matching with *T. durum* transcripts divided by plant species. (XLSX 10 kb)

Additional file 15: **Figure S5.** Functional analysis of transcripts with > 90% coverage and > 50% identity. Number of sequences with the corresponding A) Molecular Function B) Biological Process C) Cellular Component. (PDF 24 kb)

Additional file 16: **Figure S6.** Functional analysis of transcripts with > 50% coverage and > 30% identity. Number of sequences with the corresponding A) Molecular Function B) Biological Process C) Cellular Component. (PDF 24 kb)

Additional file 17 **Table S11** Significant differential expression between homoeolog genes. Under the GLM fit analysis, lines in green/brown represent genes with concordant/discordant homoeolog expression in phased blocks within the same gene. (XLSX 1605 kb)

Additional file 18: **Table S12.** Validation set. Primer pair for eight validation transcripts aligned with original homeolog-specific RNA-Seq data. (XLSX 12 kb)

Additional file 19: **Figures S7-S16.** Homeolog-specific qRT-PCR data. Results are reported for each of the thirteen varieties. Data is shown merging both leaves and roots assays but for the TRIUR3_04135, where leaves and roots assays are reported separately to visualise the opposite trend. (PDF 2674 kb)

## Abbreviations

ADT: Assay Design Tool
BUSCO: Benchmarking Universal Single-Copy Orthologs
GO: Gene Onthology
IBS: Identity By site Similarity
MAF: Minor Allele Frequency
QTL: Quantitative Trait Loci
SMRT: Single Molecule Real Time
SSR: Simple Sequence Repeats

## References

[1] Dubcovsky J, Dvořák J (2007). Genome plasticity a key factor in the success of polyploid wheat under domestication. Science..

[2] Huang Q, Börner A, Röder S, Ganal W (2002). Assessing genetic diversity of wheat (*Triticum aestivum* L.) germplasm using microsatellite markers. Theor Appl Genet.

[3] Choulet F, Wicker T, Rustenholz C, Paux E, Salse J, Leroy P, Schlub S, Le Paslier MC, Magdelenat G, Gonthier C, Couloux A, Budak H, Breen J, Pumphrey M, Liu S, Kong X, Jia J, Gut M, Brunel D, Anderson JA, Gill BS, Appels R, Keller B, Feuillet C (2010). Megabase level sequencing reveals contrasted organization and evolution patterns of the wheat gene and transposable element spaces. Plant Cell.

[4] Haudry A, Cenci A, Ravel C, Bataillon T, Brunel D, Poncet C, Hochu I, Poirier S, Santoni S, Glémin S, David J (2007). Grinding up wheat: a massive loss of nucleotide diversity since domestication. Mol Biol Evol.

[5] Dvořák J, Muehlbauer G, Feuillet C (2009). Triticeae genome structure and evolution. Genetics and genomics of the Triticeae. Plant genetics and genomics: crops and models.

[6] Daron J, Glover N, Pingault L, Theil S, Jamilloux V, Paux E, Barbe V, Mangenot S, Alberti A, Wincker P, Quesneville H, Feuillet C, Choulet F (2014). Organization and evolution of transposable elements along the bread wheat chromosome 3B. Genome Biol.

[7] Cantu D, Vanzetti LS, Sumner A, Dubcovsky M, Matvienko M, Distelfeld A, Michelmore RW, Dubcovsky J (2010). Small RNAs, DNA methylation and transposable elements in wheat. BMC Genomics.

[8] Krasileva KV, Buffalo V, Bailey P, Pearce S, Ayling S, Tabbita F, Soria M, Wang S, Akhunov E, Uauy C, Dubcovsky J, IWGS Consortium (2013). Separating homeologs by phasing in the tetraploid wheat transcriptome. Genome Biol.

[9] Duan J, Xia C, Zhao G, Jia J, Kong X (2012). Optimizing de novo common wheat transcriptome assembly using short-read RNA-Seq data. BMC Genomics.

[10] Ranwez V, Holtz Y, Sarah G, Ardisson M, Santoni S, Glémin S, Tavaud-Pirra M, David J (2013). Disentangling homeologous contigs in Allo-tetraploid assembly: application to durum wheat. BMC Bioinformatics.

[11] Mochida K, Yoshida T, Sakurai T, Ogihara Y, Shinozaki K (2009). TriFLDB: a database of clustered full-length coding sequences from Triticeae with applications to comparative grass genomics. Plant Physiol.

[12] IWGSC (The International Wheat Genome Sequencing Consortium) (2014). A chromosome-based draft sequence of the hexaploidy bread wheat (*Triticum aestivum*) genome. Science.

[13] Mascher M, Gundlach H, Himmelbach A, Beier S, Twardziok SO, Wicker T, Radchuk V, Dockter C, Hedley PE, Russell J, Bayer M, Ramsay L, Liu H, Haberer G, Zhang XQ, Zhang Q, Barrero RA, Li L, Taudien S, Groth M, Felder M, Hastie A, Šimková H, Staňková H, Vrána J, Chan S, Muñoz-Amatriaín M, Ounit R, Wanamaker S, Bolser D, Colmsee C, Schmutzer T, Aliyeva-Schnorr L, Grasso S, Tanskanen J, Chailyan A, Sampath D, Heavens D, Clissold L, Cao S, Chapman B, Dai F, Han Y, Li H, Li X, Lin C, JK MC, Tan C, Wang P, Wang S, Yin S, Zhou G, Poland JA, Bellgard MI, Borisjuk L, Houben A, Doležel J, Ayling S, Lonardi S, Kersey P, Langridge P, Muehlbauer GJ, Clark MD, Caccamo M, Schulman AH, KFX M, Platzer M, Close TJ, Scholz U, Hansson M, Zhang G, Braumann I, Spannagl M, Li C, Waugh R, Stein N (2017). A chromosome conformation capture ordered sequence of the barley genome. Nature.

[14] Avni R, Nave M, Barad O, Baruch K, Twardziok SO, Gundlach H (2017). Wild emmer genome architecture and diversity elucidate wheat evolution and domestication. Science..

[15] Trick M, Long Y, Meng J, Bancroft I (2009). Single nucleotide polymorphism (SNP) discovery in the polyploid Brassica napus using Solexa transcriptome sequencing. Plant Biotechnol J.

[16] Nagy I, Barth S, Mehenni-Ciz J, Abberton MT, Milbourne D (2013). A hybrid next generation transcript sequencing-based approach to identify allelic and homeolog-specific single nucleotide polymorphisms in allotetraploid white clover. BMC Genomics.

[17] Wang Shichen, Wong Debbie, Forrest Kerrie, Allen Alexandra, Chao Shiaoman, Huang Bevan E., Maccaferri Marco, Salvi Silvio, Milner Sara G., Cattivelli Luigi, Mastrangelo Anna M., Whan Alex, Stephen Stuart, Barker Gary, Wieseke Ralf, Plieske Joerg, Lillemo Morten, Mather Diane, Appels Rudi, Dolferus Rudy, Brown-Guedira Gina, Korol Abraham, Akhunova Alina R., Feuillet Catherine, Salse Jerome, Morgante Michele, Pozniak Curtis, Luo Ming-Cheng, Dvorak Jan, Morell Matthew, Dubcovsky Jorge, Ganal Martin, Tuberosa Roberto, Lawley Cindy, Mikoulitch Ivan, Cavanagh Colin, Edwards Keith J., Hayden Matthew, Akhunov Eduard (2014). Characterization of polyploid wheat genomic diversity using a high-density 90 000 single nucleotide polymorphism array. Plant Biotechnology Journal.

[18] Maccaferri M, Sanguineti MC, Corneti S, Ortega JLA, Salem MB, Bort J, DeAmbrogio E, Garcia del Moral LF, Demontis A, El-Ahmed A, Maalouf F, Machlab H, Martos V, Moragues M, Motawaj J, Nachit M, Nserallah N, Ouabbou H, Royo C, Slama A, Tuberosa R (2008). Quantitative trait loci for grain yield and adaptation of durum wheat (*Triticum durum* Desf.) across a wide range of water availability. Genetics.

[19] Maccaferri M, Sanguineti MC, Noli E, Tuberosa R (2005). Population structure and long-range linkage disequilibrium in a durum wheat elite collection. Mol Breed.

[20] Grabherr MG, Haas BJ, Yassour M, Levin JZ, Thompson DA, Amit I, Adiconis X, Fan L, Raychowdhury R, Zeng Q, Chen Z, Mauceli E, Hacohen N, Gnirke A, Rhind N, di Palma F, Birren BW, Nusbaum C, Lindblad-Toh K, Friedman N, Regev A (2011). Full-length transcriptome assembly from RNA-Seq data without a reference genome. Nat Biotechnol.

[21] Gibbons JG, Janson EM, Hittinger CT, Johnston M, Abbot P, Rokas A (2009). Benchmarking next-generation transcriptome sequencing for functional and evolutionary genomics. Mol Biol Evol.

[22] Zhao QY, Wang Y, Kong YM, Luo D, Li X, Hao P (2011). Optimizing de novo transcriptome assembly from short-read RNA-Seq data: a comparative study. BMC Bioinformatics.

[23] Ogihara Y, Mochida K, Kawaura K, Murai K, Seki M, Kamiya A, Shinozaki K, Carninci P, Hayashizaki Y, Shin-I T, Kohara Y, Yamazaki Y (2004). Construction of a full-length cDNA library from young spikelets of hexaploid wheat and its characterization by large-scale sequencing of expressed sequence tags. Genes Genet Syst.

[24] Higgins J, Magusin A, Trick M, Fraser F, Bancroft I (2012). Use of mRNA-seq to discriminate contributions to the transcriptome from the constituent genomes of the polyploid crop species Brassica napus. BMC Genomics.

[25] Liu JJ, Sniezko RA, Sturrock RN, Chen H (2014). Western white pine SNP discovery and high-throughput genotyping for breeding and conservation applications. BMC Plant Biol.

[26] He Z, Cheng F, Li Y, Wang X, Parkin IA, Chalhoub B, Liu S, Bancroft I (2015). Construction of Brassica a and C genome-based ordered pan-transcriptomes for use in rapeseed genomic research. Data Brief.

[27] Pingault L, Choulet F, Alberti A, Glover N, Wincker P, Feuillet C, Paux E (2015). Deep transcriptome sequencing provides new insights into the structural and functional organization of the wheat genome. Genome Biol.

[28] Dong L, Liu H, Zhang J, Yang S, Kong G, Chu JS, Chen N, Wang D (2015). Single-molecule real-time transcript sequencing facilitates common wheat genome annotation and grain transcriptome research. BMC Genomics.

[29] Sim Sung-Chur, Van Deynze Allen, Stoffel Kevin, Douches David S., Zarka Daniel, Ganal Martin W., Chetelat Roger T., Hutton Samuel F., Scott John W., Gardner Randolph G., Panthee Dilip R., Mutschler Martha, Myers James R., Francis David M. (2012). High-Density SNP Genotyping of Tomato (Solanum lycopersicum L.) Reveals Patterns of Genetic Variation Due to Breeding. PLoS ONE.

[30] Hirsch CN, Foerster JM, Johnson JM, Sekhon RS, Muttoni G, Vaillancourt B, Peñagaricano F, Lindquist E, Pedraza MA, Barry K, de Leon N, Kaeppler SM, Buell CR (2014). Insights into the maize pan-genome and pan-transcriptome. Plant Cell.

[31] Goettel W, Xia E, Upchurch R, Wang ML, Chen P, An YQ (2014). Identification and characterization of transcript polymorphisms in soybean lines varying in oil composition and content. BMC Genomics.

[32] Kim JE, Oh SK, Lee JH, Lee BM, Jo SH (2014). Genome-wide SNP calling using next generation sequencing data in tomato. Mol Cells.

[33] Ren J, Chen L, Sun D, You FM, Wang J, Peng Y, Nevo E, Beiles A, Sun D, Luo MC, Peng J (2013). SNP-revealed genetic diversity in wild emmer wheat correlates with ecological factors. BMC Evol Biol.

[34] Faris JD, Zhang Q, Chao S, Zhang Z, Xu SS (2014). Analysis of agronomic and domestication traits in a durum × cultivated emmer wheat population using a high-density single nucleotide polymorphism-based linkage map. Theor Appl Genet.

[35] Maccaferri M, Ricci A, Salvi S, Milner SG, Noli E, Martelli PL, Casadio R, Akhunov E, Scalabrin S, Vendramin V, Ammar K, Blanco A, Desiderio F, Distelfeld A, Dubcovsky J, Fahima T, Faris J, Korol A, Massi A, Mastrangelo AM, Morgante M, Pozniak C, N’Diaye A, Xu S, Tuberosa R (2015). A high-density, SNP-based consensus map of tetraploid wheat as a bridge to integrate durum and bread wheat genomics and breeding. Plant Biotechnol J.

[36] Dvorak J, Akhunov ED, Akhunov AR, Deal KR, Luo MC (2006). Molecular characterization of a diagnostic DNA marker for domesticated tetraploid wheat provides evidence for gene flow from wild tetraploid wheat to exaploidy wheat. Mol Biol Evol.

[37] Thuillet AC, Bataillon T, Poirier S, Santoni S, David JL (2005). Estimation of long-term effective population sizes through the history of durum wheat using microsatellite data. Genetics..

[38] Ling HQ, Zhao S, Liu D, Wang J, Sun H, Zhang C, Fan H, Li D, Dong L, Tao Y, Gao C, Wu H, Li Y, Cui Y, Guo X, Zheng S, Wang B, Yu K, Liang Q, Yang W, Lou X, Chen J, Feng M, Jian J, Zhang X, Luo G, Jiang Y, Liu J, Wang Z, Sha Y, Zhang B, Wu H, Tang D, Shen Q, Xue P, Zou S, Wang X, Liu X, Wang F, Yang Y, An X, Dong Z, Zhang K, Zhang X, Luo MC, Dvořák J, Tong Y, Wang J, Yang H, Li Z, Wang D, Zhang A, Wang J (2013). Draft genome of the wheat A-genome progenitor *Triticum urartu*. Nature..

[39] Ling HQ, Ma B, Shi X, Liu H, Dong L, Sun H, Cao Y, Gao Q, Zheng S, Li Y, Yu Y, Du H, Qi M, Li Y, Lu H, Yu H, Cui Y, Wang N, Chen C, Wu H, Zhao Y, Zhang J, Li Y, Zhou W, Zhang B, Hu W, van Eijk MJT, Tang J, Witsenboer HMA, Zhao S, Li Z, Zhang A, Wang D, Liang C (2018). Genome sequence of the progenitor of wheat a subgenome *Triticum urartu*. Nature. 2018. Nature..

[40] Luo MC, Gu YQ, Puiu D, Wang H, Twardziok SO, Deal KR, Huo N, Zhu T, Wang L, Wang Y, PE MG, Liu S, Long H, Ramasamy RK, Rodriguez JC, Van SL YL, Wang Z, Xia Z, Xiao L, Anderson OD, Ouyang S, Liang Y, Zimin AV, Pertea G, Qi P, Bennetzen JL, Dai X, Dawson MW, Müller HG, Kugler K, Rivarola-Duarte L, Spannagl M, KFX M, Lu FH, Bevan MW, Leroy P, Li P, You FM, Sun Q, Liu Z, Lyons E, Wicker T, Salzberg SL, Devos KM, Dvořák J (2017). Genome sequence of the progenitor of the wheat D genome *Aegilops tauschii*. Nature.

[41] Degner JF, Marioni JC, Pai AA, Pickrell JK, Nkadori E, Gilad Y, Pritchard JK (2009). Effect of read-mapping biases on detecting allele-specific expression from RNA-sequencing data. Bioinformatics..

[42] Harper AL, Trick M, He Z, Clissold L, Fellgett A, Griffiths S, Bancroft I (2016). Genome distribution of differential homoeologue contributions to leaf gene expression in bread wheat. Plant Biotechnol J.

[43] Zadoks JC, Chang TT, Konzak CF (1974). A decimal code for the growth stages of cereals. Weed Res.

[44] Vezzi F, Del Fabbro C, Tomescu AI, Policriti A (2012). rNA: a fast and accurate short reads numerical aligner. Bioinformatics..

[45] Zerbino DR, Birney E (2008). Velvet: algorithms for de novo short read assembly using de Bruijn graphs. Genome Res.

[46] Schulz Marcel H., Zerbino Daniel R., Vingron Martin, Birney Ewan (2012). Oases: robust de novo RNA-seq assembly across the dynamic range of expression levels. Bioinformatics.

[47] Simão FA, Waterhouse RM, Ioannidis P, Kriventseva EV, Zdobnov EM (2015). BUSCO: assessing genome assembly and annotation completeness with single-copy orthologs. Bioinformatics..

[48] Vincent J, Dai Z, Ravel C, Choulet F, Mouzeyar MS, Bouzidi F, Agier M, Martre P (2013). dbWFA: a web-based database for functional annotation of *Triticum aestivum* transcripts. Database.

[49] Herrero J, Muffato M, Beal K, Fitzgerald S, Gordon L, Pignatelli M, Vilella AJ, Searle SM, Amode R, Brent S, Spooner W, Kulesha E, Yates A, Flicek P. Ensembl comparative genomics resources. Database.2016; bav053. http://www.ensembl.org/biomart/martview. 10.1093/database/baw053 PMC4852398 27141089

[50] Piovesan D., Luigi Martelli P., Fariselli P., Zauli A., Rossi I., Casadio R. (2011). BAR-PLUS: the Bologna Annotation Resource Plus for functional and structural annotation of protein sequences. Nucleic Acids Research.

[51] Piovesan Damiano, Profiti Giuseppe, Martelli Pier Luigi, Fariselli Piero, Casadio Rita (2013). Extended and Robust Protein Sequence Annotation over Conservative Nonhierarchical Clusters. ACM Journal on Emerging Technologies in Computing Systems.

[52] Conesa A, Götz S, García-Gómez JM, Terol J, Talón M, Robles M (2005). Blast2go: a universal tool for annotation, visualization and analysis in functional genomics research. Bioinformatics..

[53] Langmead B, Salzberg S (2012). Fast gapped-read alignment with bowtie 2. Nat Methods.

[54] DePristo M, Banks E, Poplin R, Garimella K, Maguire J, Hartl C, Philippakis A, del Angel G, Rivas MA, Hanna M, McKenna A, Fennell T, Kernytsky A, Sivachenko A, Cibulskis K, Gabriel S, Altshuler D, Daly M (2011). A framework for variation discovery and genotyping using next-generation DNA sequencing data. Nat Genet.

[55] Van der Auwera GA, Carneiro M, Hartl C, Poplin R, del Angel G, Levy-Moonshine A, Jordan T, Shakir K, Roazen D, Thibault J, Banks E, Garimella K, Altshuler D, Gabriel S, DePristo M (2013). From FastQ data to high-confidence variant calls: the genome analysis toolkit best practices pipeline. Curr Protoc Bioinformatics.

[56] Bansal V, Bafna V (2008). HapCUT: an efficient and accurate algorithm for the haplotype assembly problem. Bioinformatics..

[57] Li H, Durbin R (2009). Fast and accurate short read alignment with burrows-wheeler transform. Bioinformatics.

[58] Anders Simon, Huber Wolfgang (2010). Differential expression analysis for sequence count data. Genome Biology.

[59] Anders S. Package ‘DESeq’. 2018. http://bioconductor.org/packages/release/bioc/manuals/DESeq/man/DESeq.pdf. Accessed 10 Mar 2018.

[60] Canè MA, Maccaferri M, Nazemi G, Salvi S, Francia R, Colalongo C (2014). Association mapping for root architectural traits in durum wheat seedlings as related to agronomic performance. Mol Breed.

[61] International Wheat Genome Sequencing Consortium (IWGSC) (2018). Shifting the limits in wheat research and breeding using a fully annotated reference genome. Science.

[62] Koressaar T, Remm M (2007). Enhancements and modifications of primer design program Primer3. Bioinformatics.

[63] Untergasser A, Cutcutache I, Koressaar T, Ye J, Faircloth BC, Remm M (2012). Primer3--new capabilities and interfaces. Nucleic Acids Res.

[64] Giménez MJ, Pistón F, Atienza SG (2011). Identification of suitable reference genes for normalization of qPCR data in comparative transcriptomics analyses in the Triticeae. Planta..

[65] Zhang W, Chen S, Abate Z, Nirmala J, Rouse MN, Dubcovsky J (2017). Identification and characterization of Sr13, a tetraploid wheat gene that confers resistance to the Ug99 stem rust race group. Proc Natl Acad Sci U S A.

