# Supplementary figures and images for: Genomic tools for durum wheat breeding: de novo assembly of Svevo transcriptome and SNP discovery in elite germplasm
Source: BMC Genomics. 2019 Apr 10;20:278. doi: 10.1186/s12864-019-5645-x (PMC6456968; doi:10.1186/s12864-019-5645-x)

# Assembly contigs size distribution

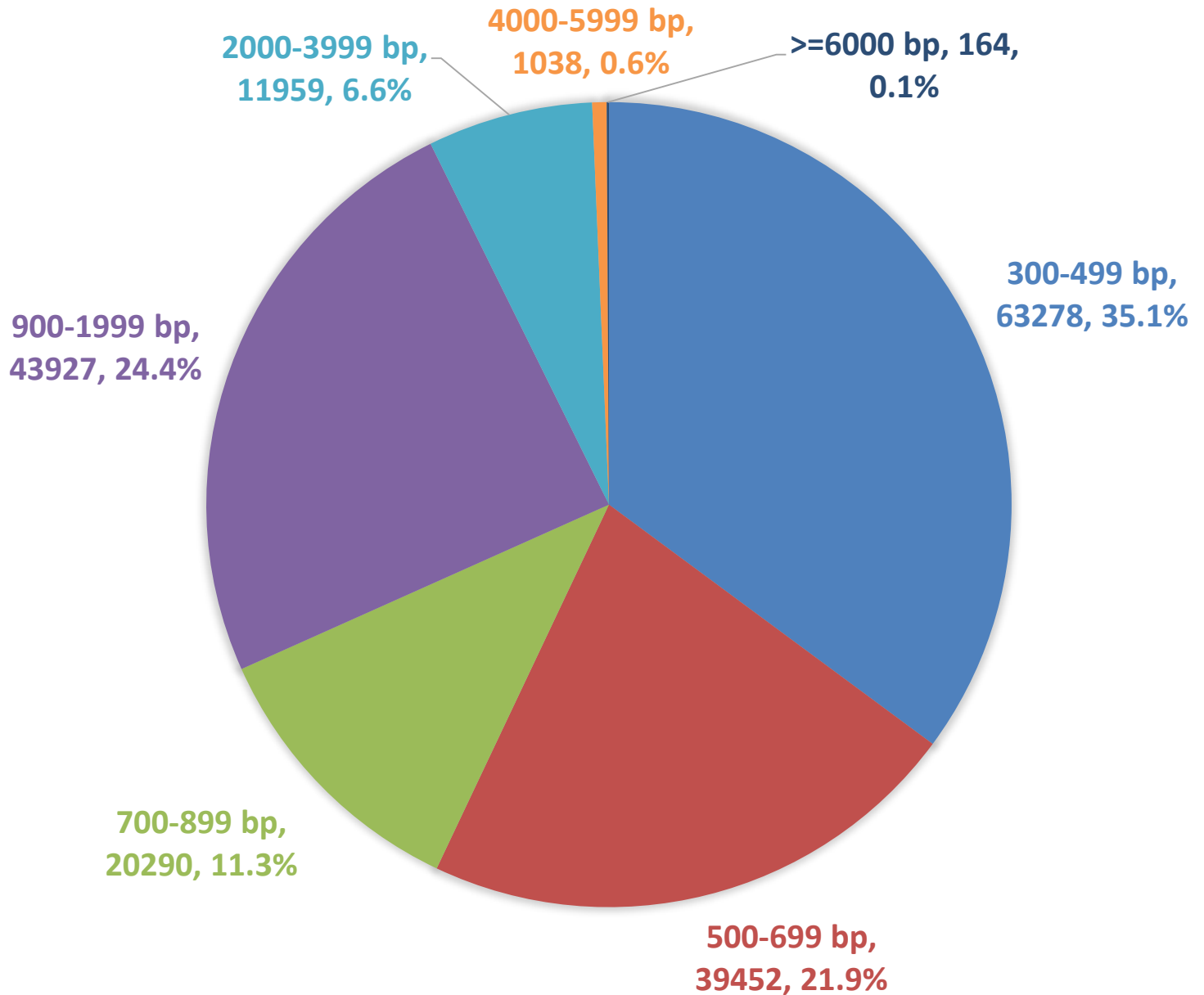

Supplement: Supplementary file 4 — Figure S1. Assembly contigs size distribution. Contigs size distribution in the selected assembly (CLC with k-mer size = 64). (PDF 113 kb) [file 12864_2019_5645_MOESM4_ESM.pdf]

A

## Number of contigs - CLC

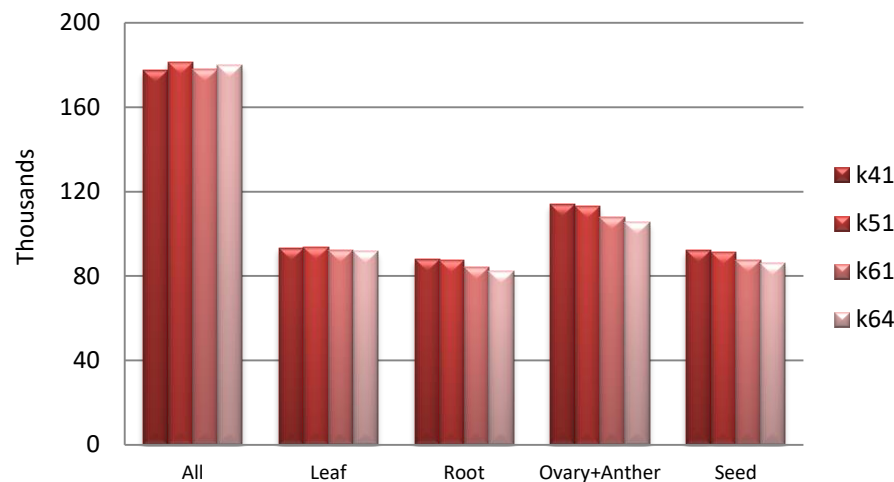

B

## N50 - CLC

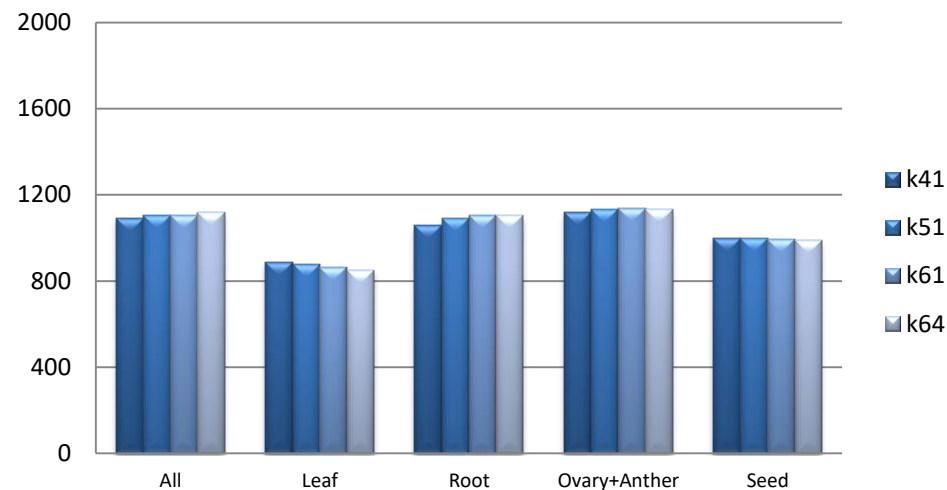

C

## Number of contigs - VELVET

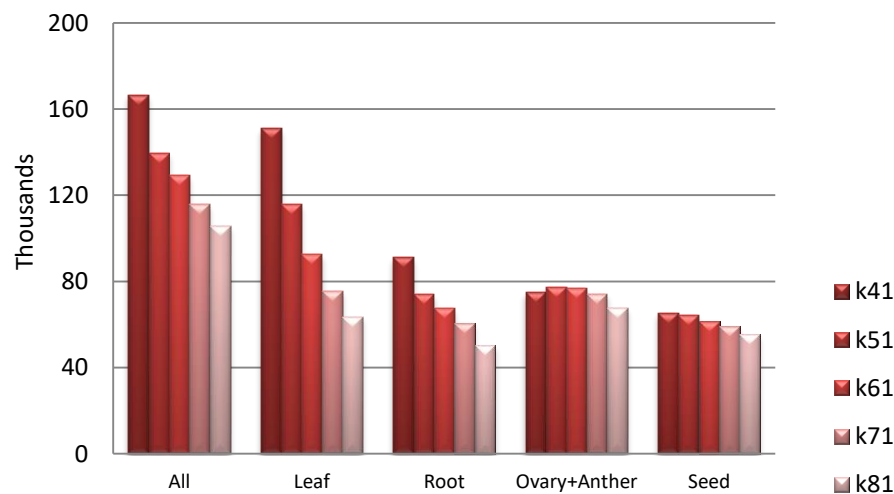

D

## N50 - VELVET

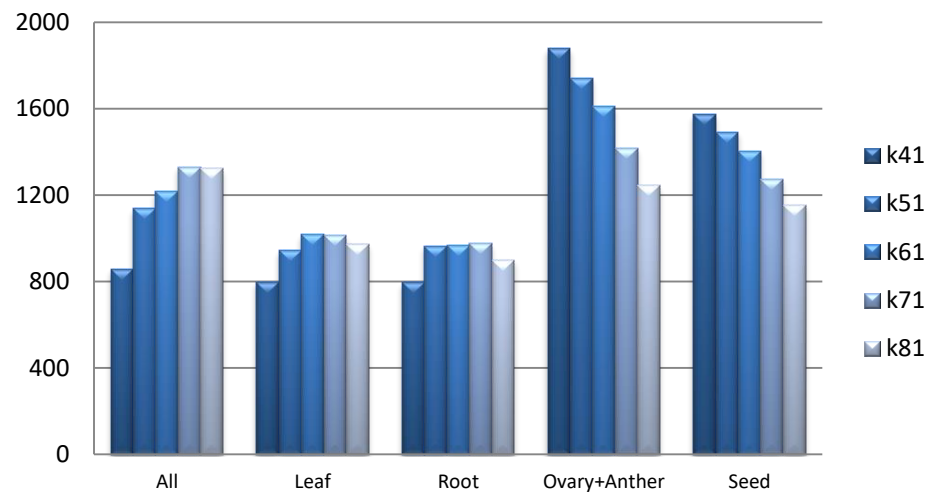

Supplement: Supplementary file 5 — Figure S2. Evaluation of de novo assemblies. Comparison at different k-mer values (i.e. k41, k51, k61, k64) and with different tools among different assemblies. (A) and (B) refer to assemblies computed with CLC and indicate, respectively, number of contigs and N50. (C) and (D) refer to Velvet-Oases assemblies. (PDF 601 kb) [file 12864_2019_5645_MOESM5_ESM.pdf]

>90% protein coverage, > 50% identity

BioMart

BAR+

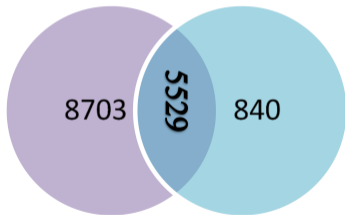

>50% protein coverage, > 30% identity

BioMart

BAR+

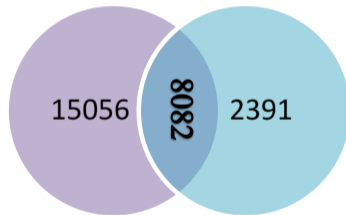

Supplement: Supplementary file 12 — Figure S4. Merged BioMart and BAR+ annotations. Venn diagram of transcript annotations with Ensembl and BAR+. On the left, at > 90% protein coverage and > 50% identity a total of 15,072 transcripts were annotated; while, on the right, at > 50% protein coverage and > 30% identity a total of 25,529 transcripts were annotated. (PDF 399 kb) [file 12864_2019_5645_MOESM12_ESM.pdf]

A

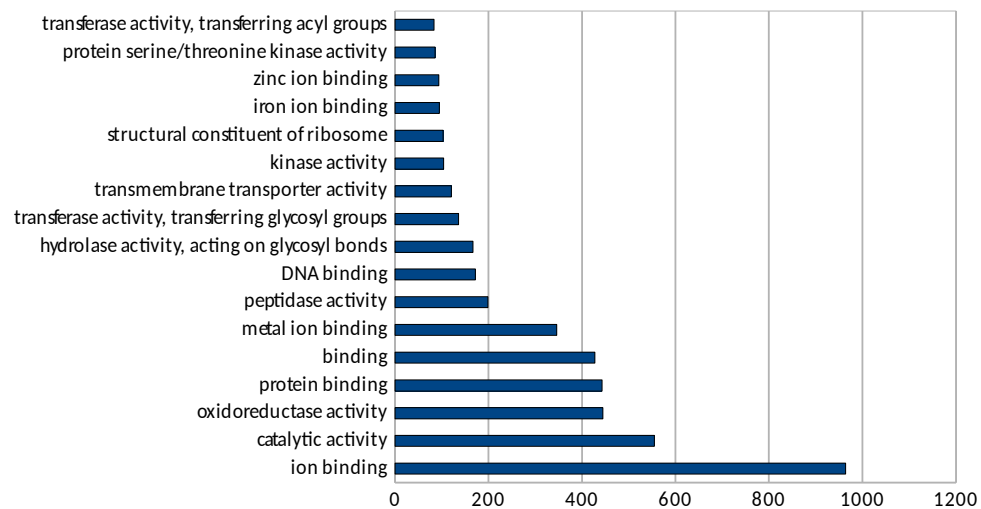

C

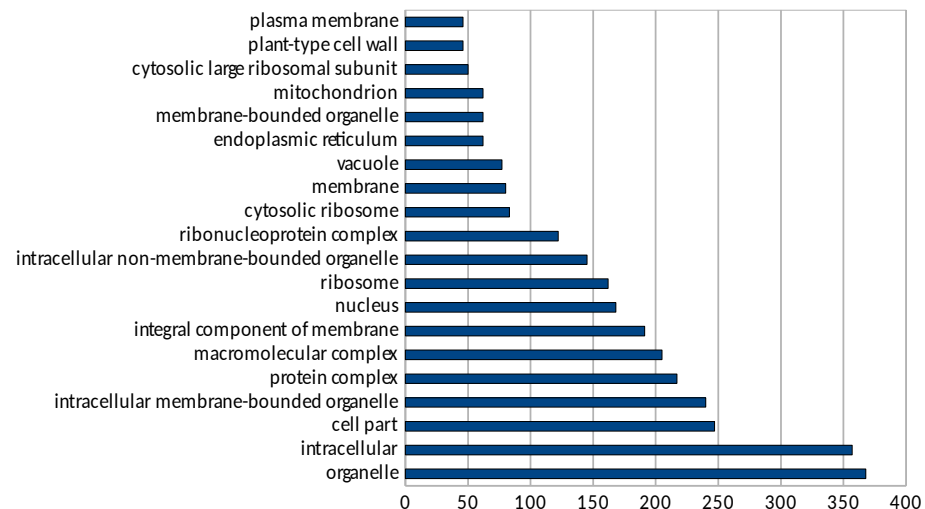

B

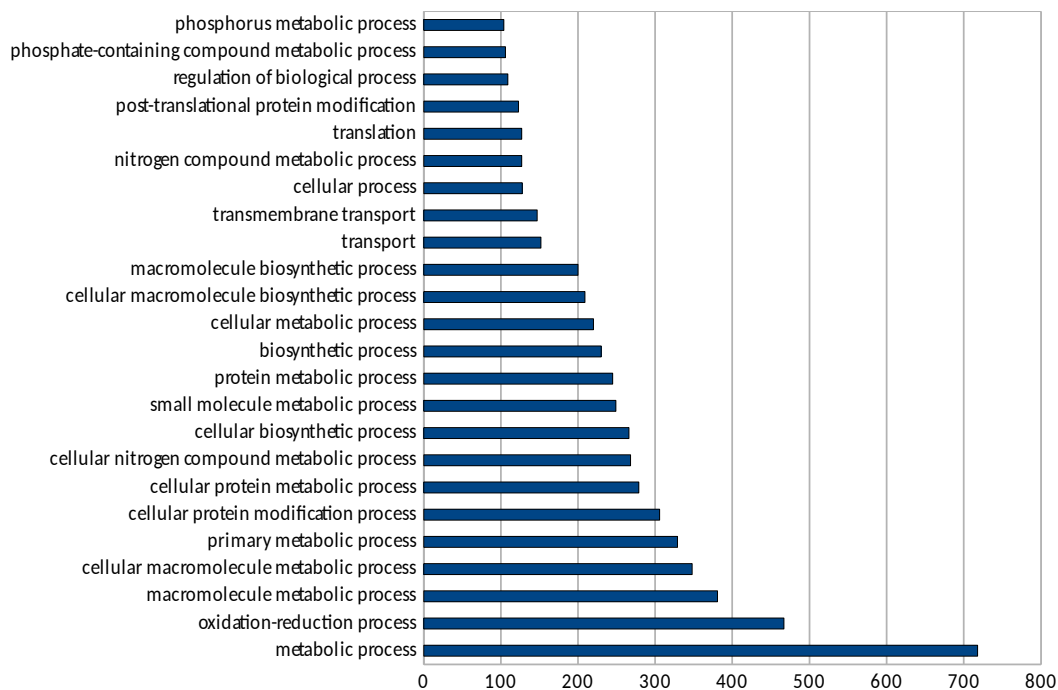

Supplement: Supplementary file 15 — Figure S5. Functional analysis of transcripts with > 90% coverage and > 50% identity. Number of sequences with the corresponding A) Molecular Function B) Biological Process C) Cellular Component. (PDF 24 kb) [file 12864_2019_5645_MOESM15_ESM.pdf]

A

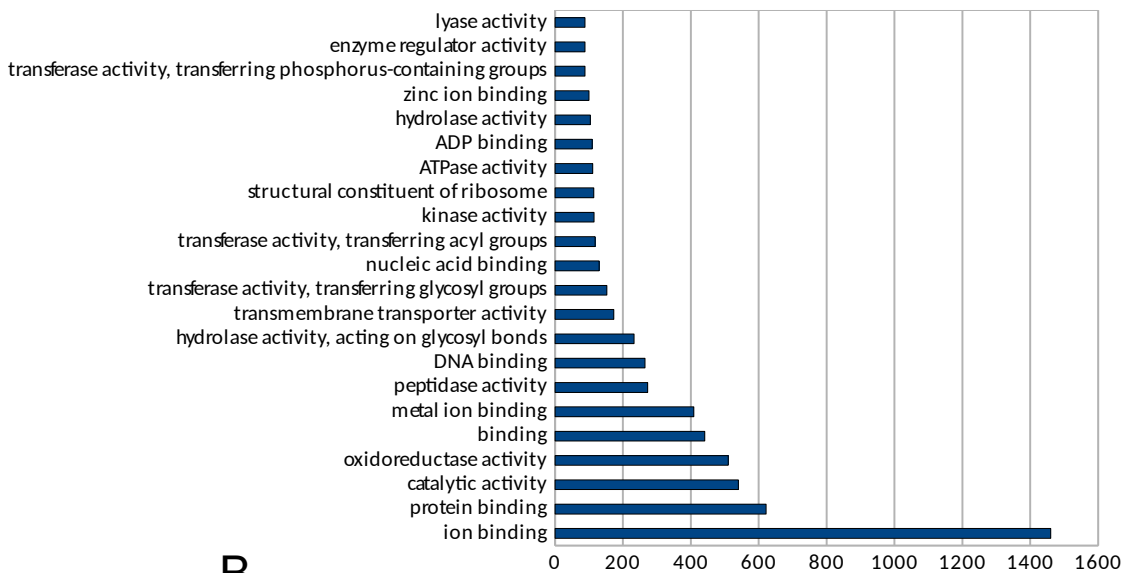

B

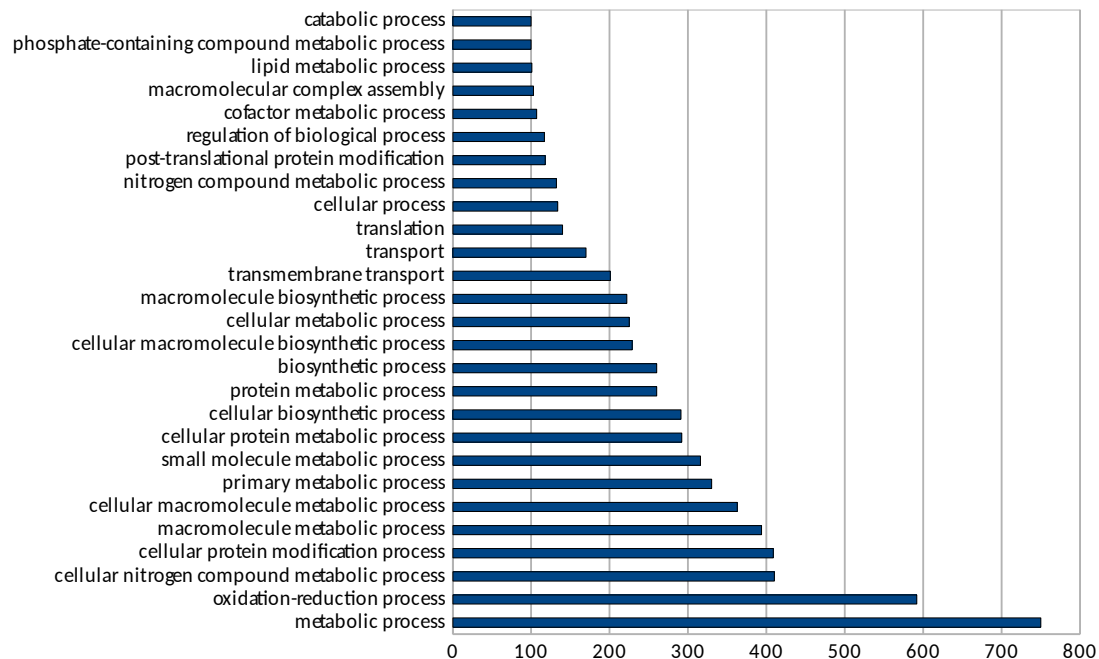

C

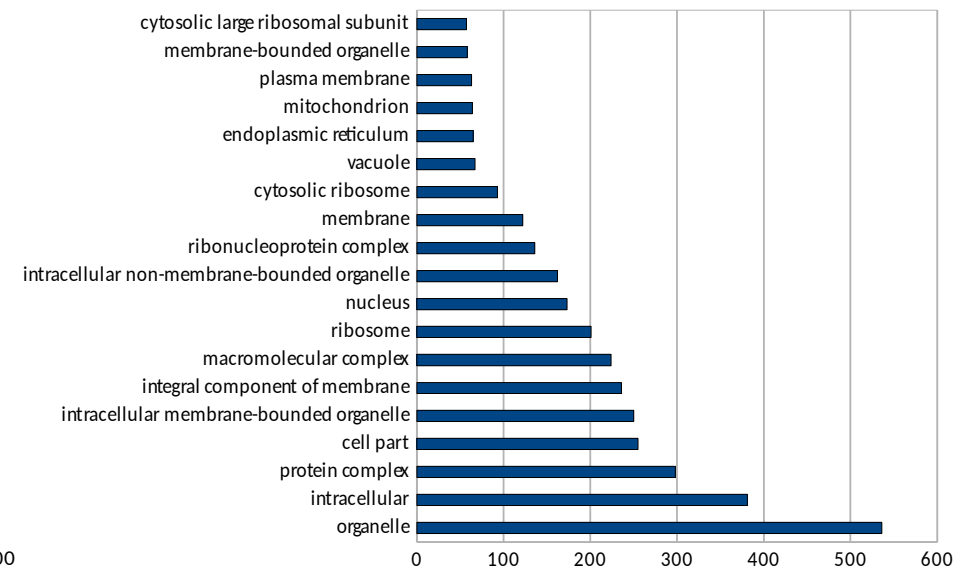

Supplement: Supplementary file 16 — Figure S6. Functional analysis of transcripts with > 50% coverage and > 30% identity. Number of sequences with the corresponding A) Molecular Function B) Biological Process C) Cellular Component. (PDF 24 kb) [file 12864_2019_5645_MOESM16_ESM.pdf]

# TRIUR3\_04135\_Leaf

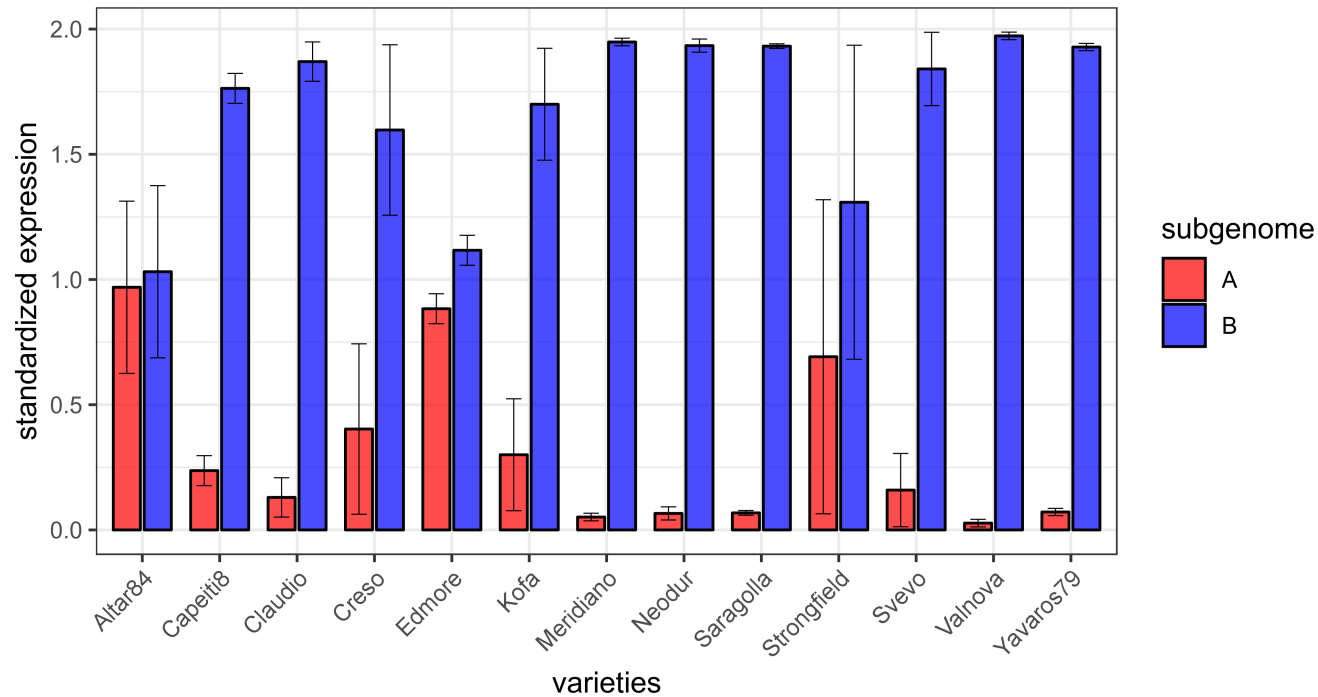

# TRIUR3\_04135\_Root

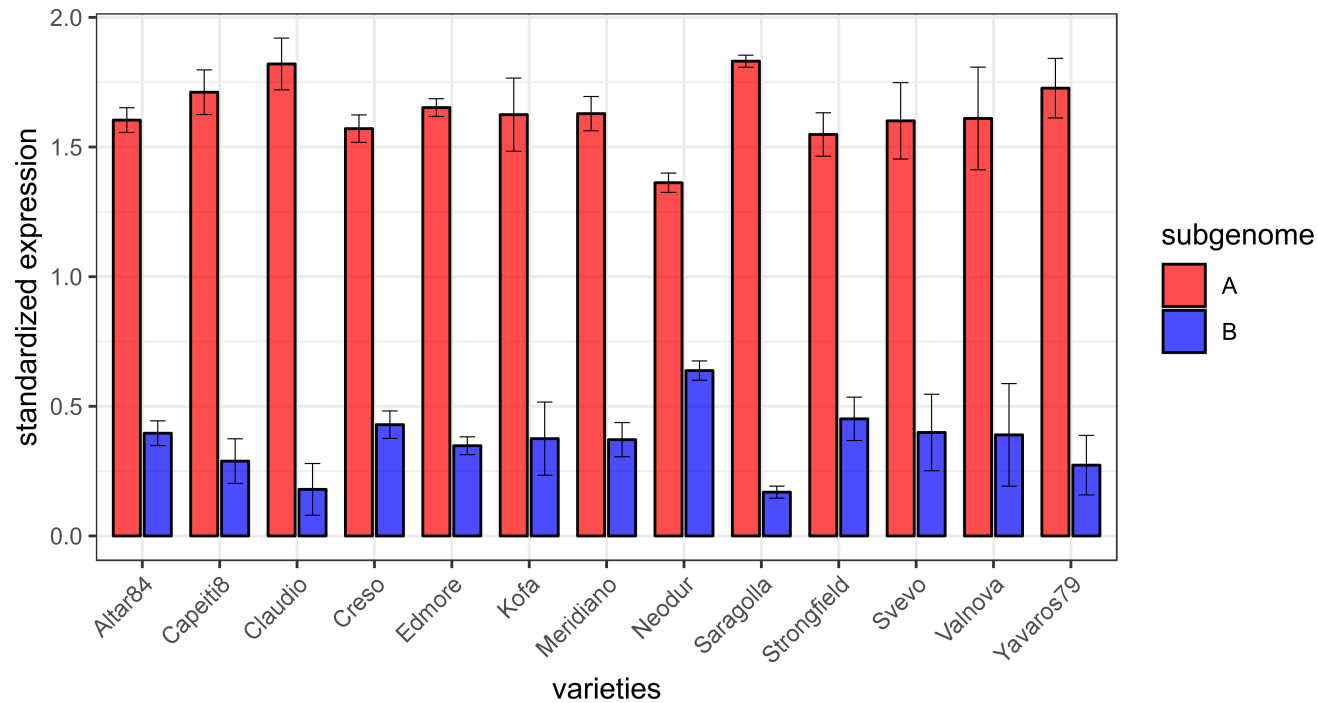

# TRIUR3\_14772\_L+R

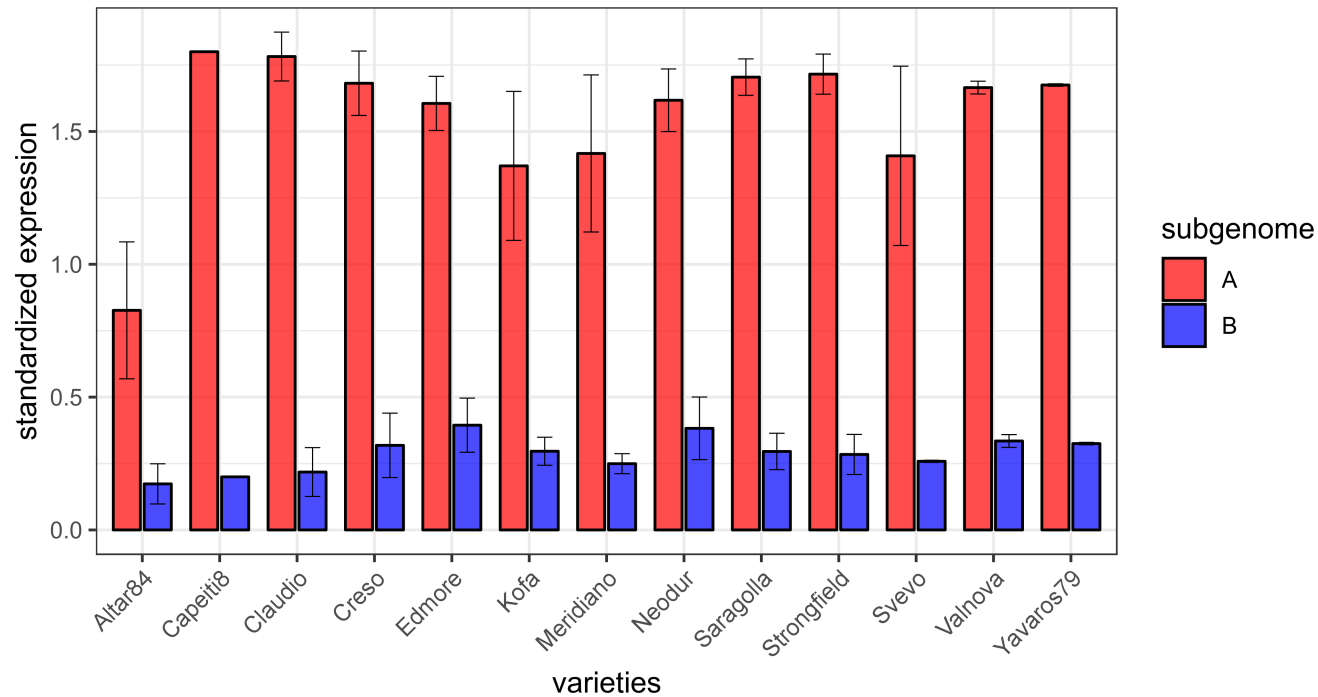

# TRIUR3\_09011\_L+R

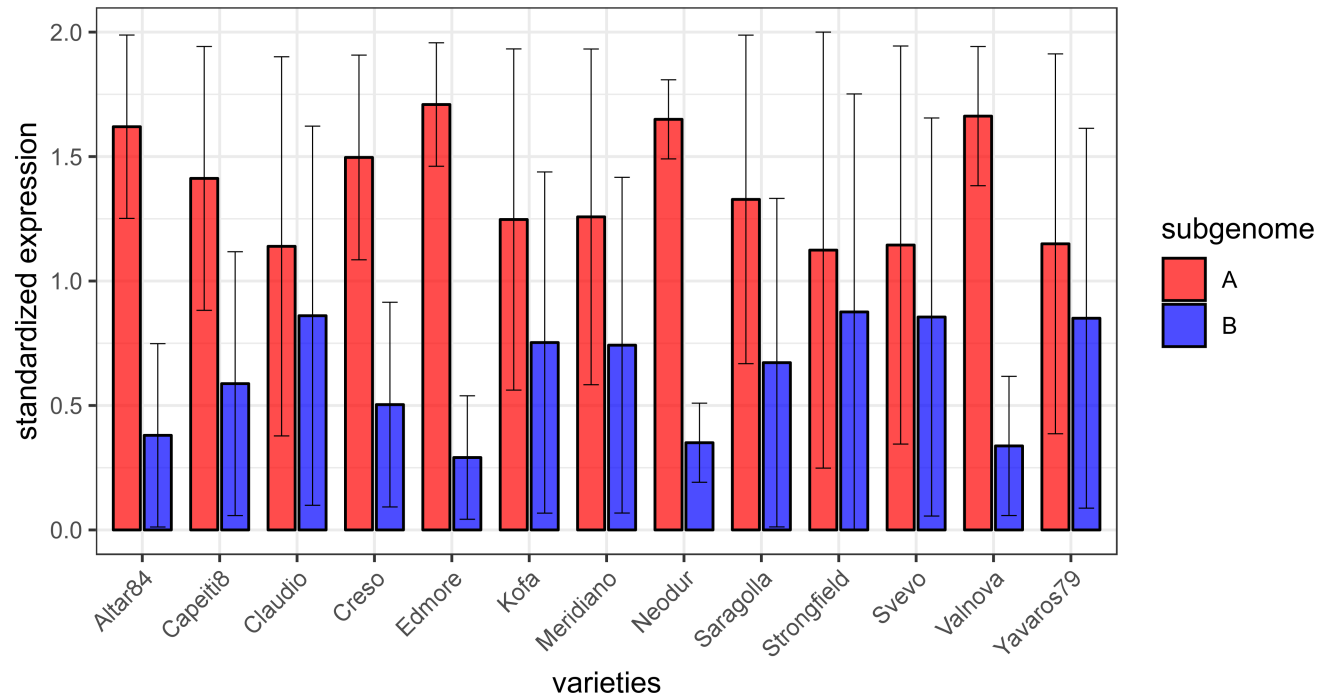

# TRIUR3\_06137\_L+R

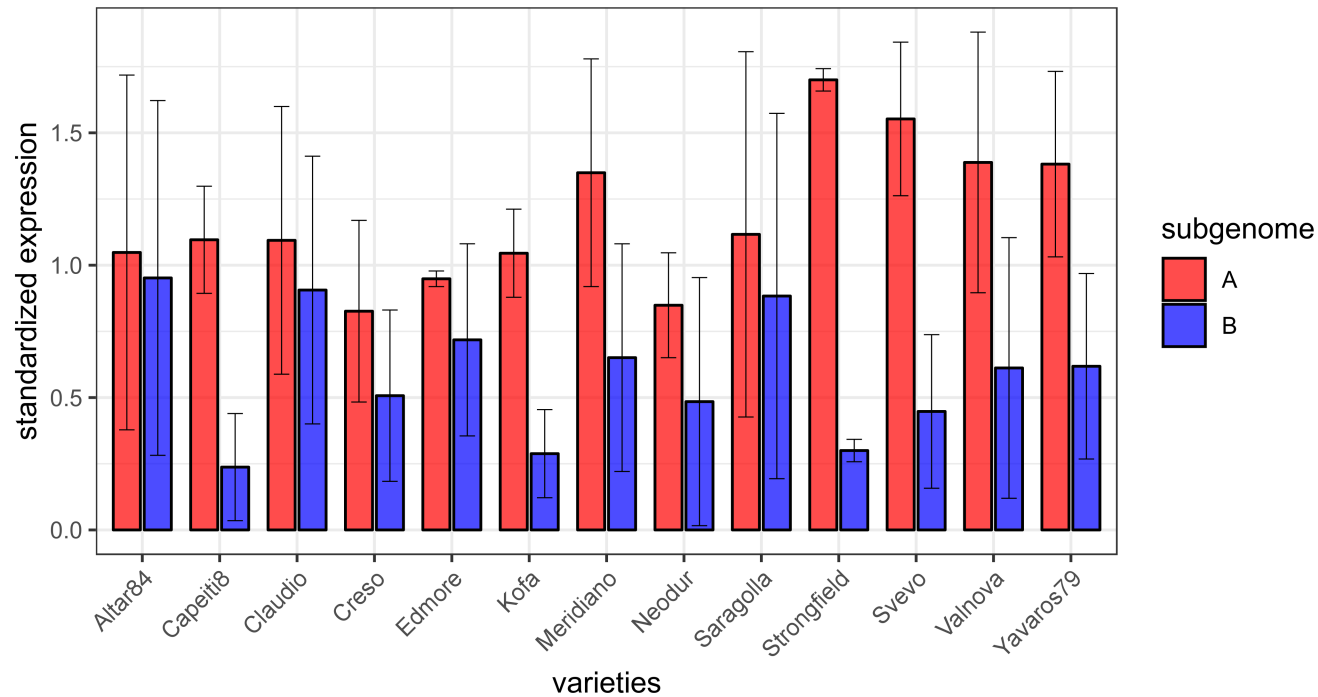

# TRIUR3\_05979\_L+R

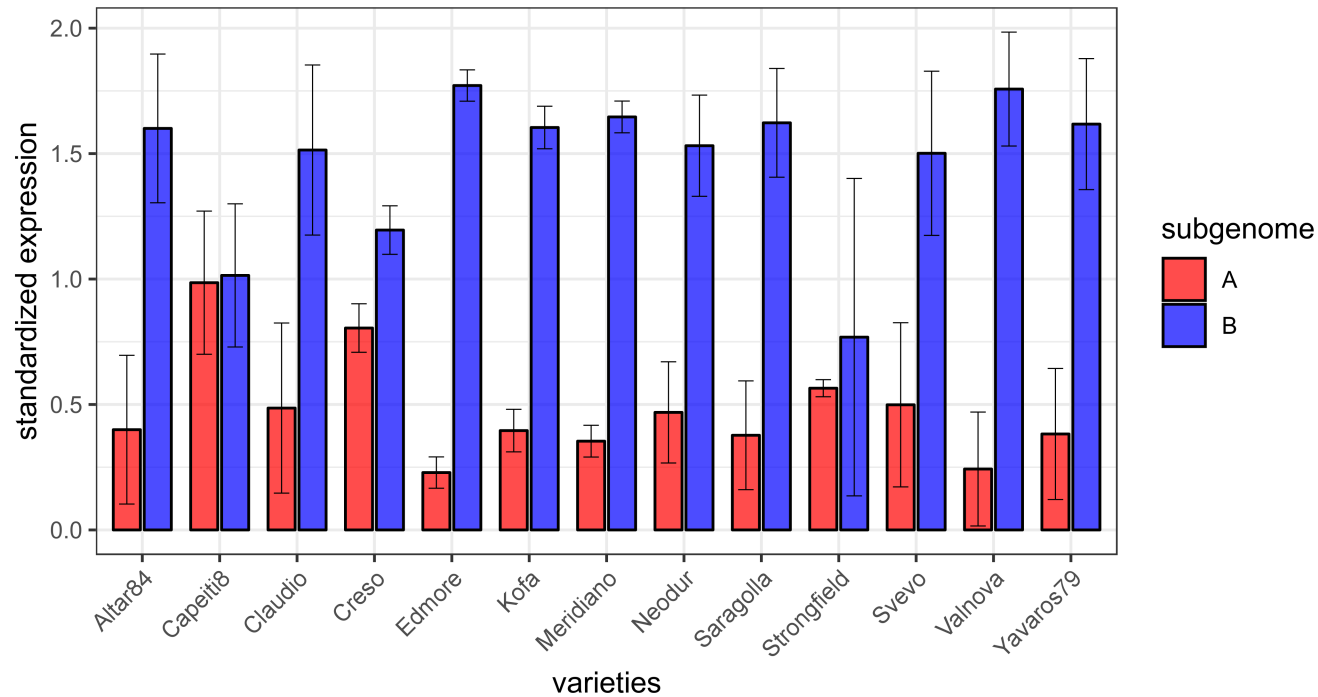

# TRIUR3\_07762\_L+R

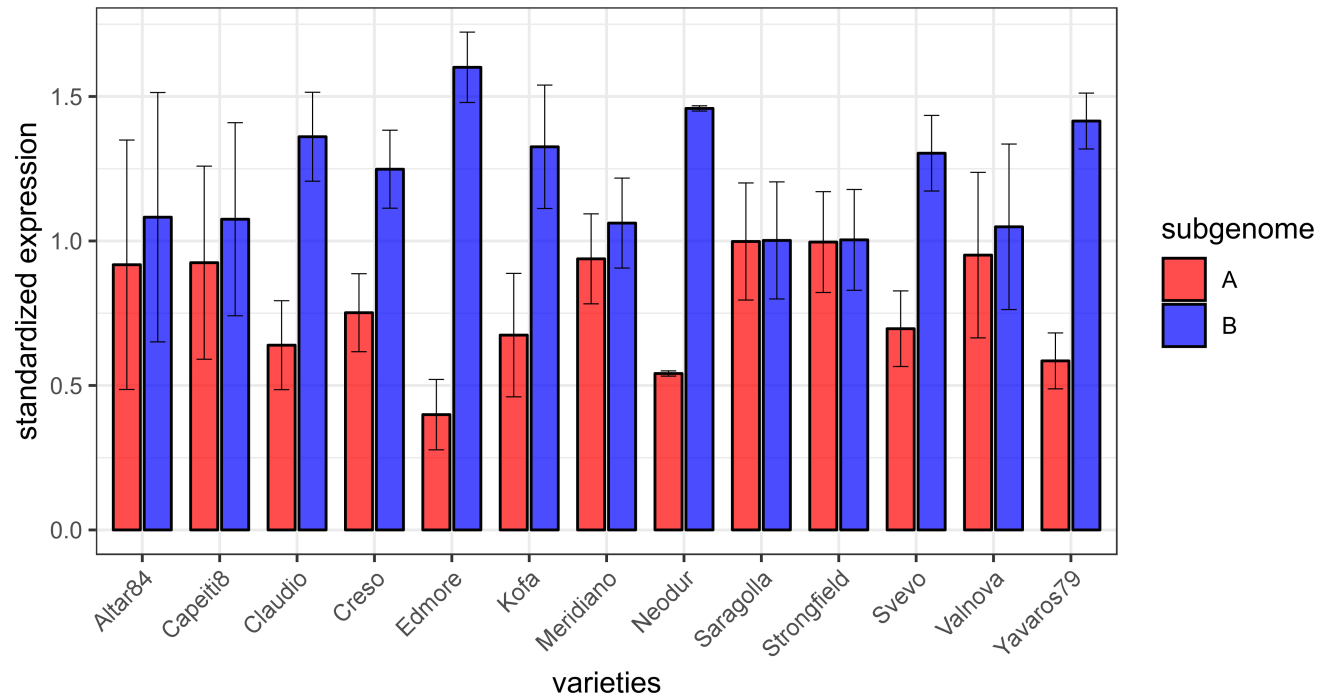

# TRIUR3\_15361\_L+R

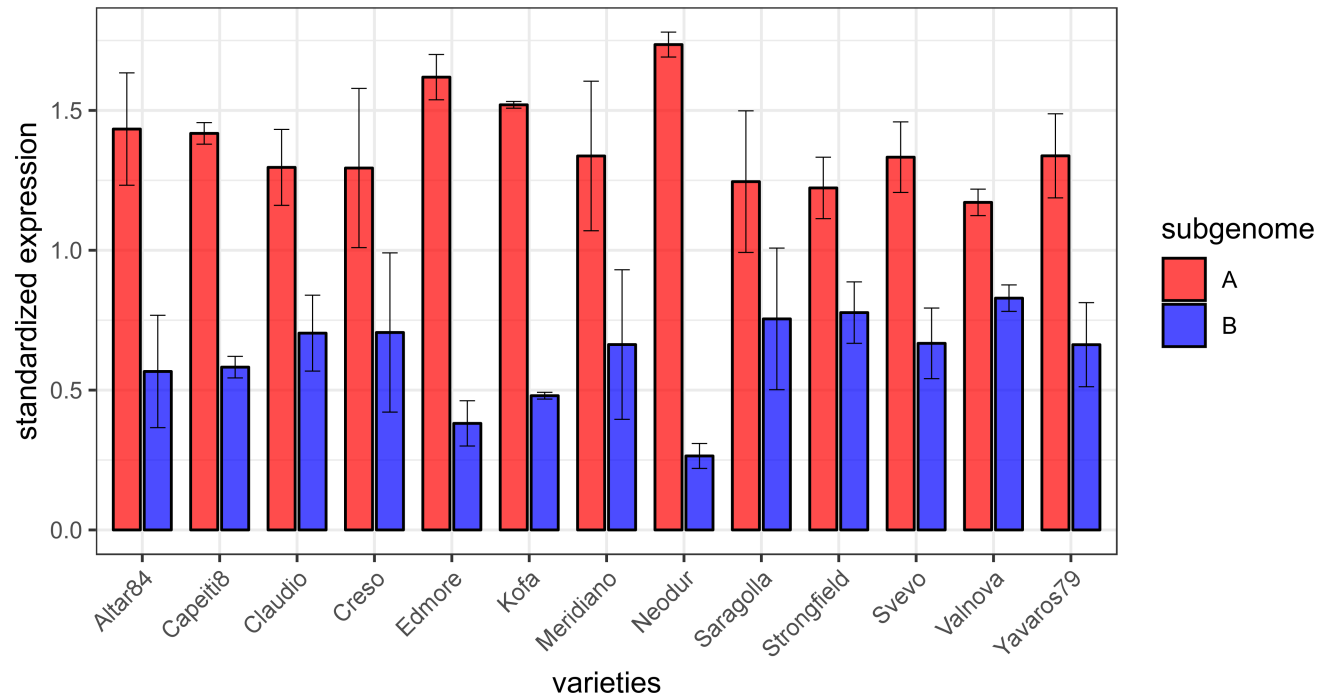

# TRIUR3\_14342\_L+R

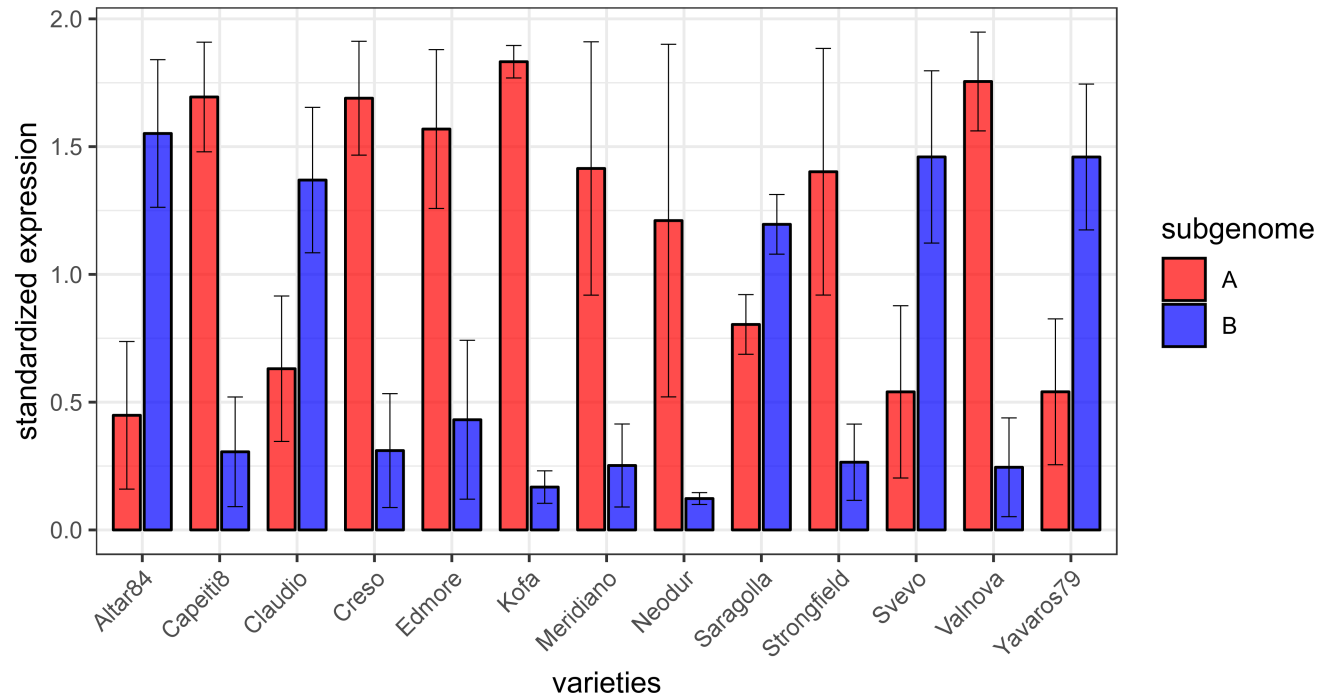

Supplement: Supplementary file 19 — Figures S7-S16. Homeolog-specific qRT-PCR data. Results are reported for each of the thirteen varieties. Data is shown merging both leaves and roots assays but for the TRIUR3_04135, where leaves and roots assays are reported separately to visualise the opposite trend. (PDF 2674 kb) [file 12864_2019_5645_MOESM19_ESM.pdf]
