# Supplementary material for: Genomic tools for durum wheat breeding: de novo assembly of Svevo transcriptome and SNP discovery in elite germplasm
Source: BMC Genomics. 2019 Apr 10;20:278. doi: 10.1186/s12864-019-5645-x (PMC6456968; doi:10.1186/s12864-019-5645-x)
Supplement: Supplementary file 6 — Figure S3. Validation of assemblies. The best assembly was validated versus two datasets: (A) full-length cDNAs [11, 23]; (B) Triticum aestivum chromosome 3B genes. Bars represent the percentage of genes reconstructed at least in 80% of their length. Different colors represent the number of different contigs necessary to reconstruct the genes. (PDF 104 kb) [file 12864_2019_5645_MOESM6_ESM.pdf]

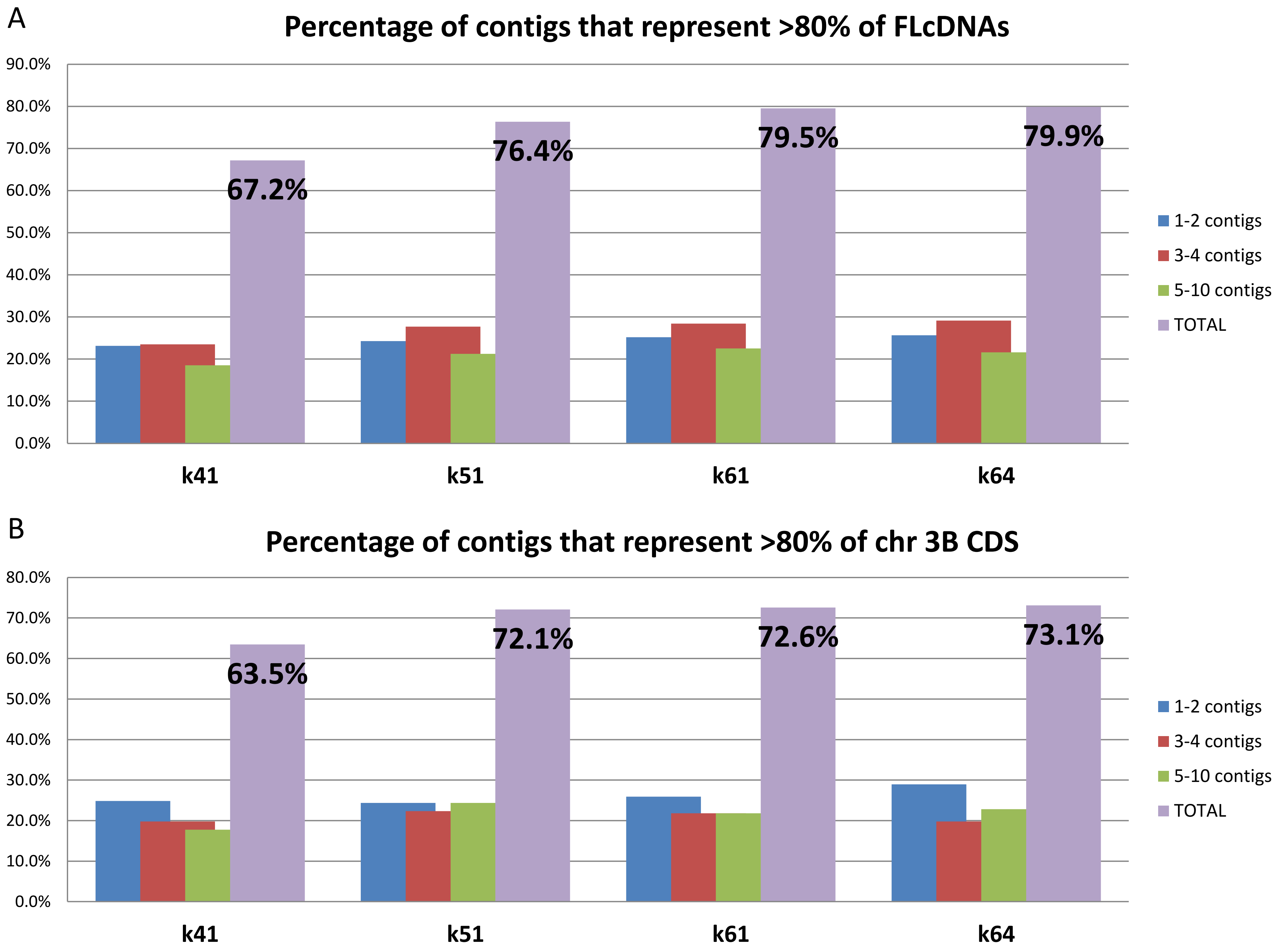
A
Percentage of contigs that represent >80% of FLcDNAs
90.0%
80.0%
70.0%
60.0%
50.0%
40.0%
30.0%
20.0%
10.0%
0.0%
67.2%
76.4%
79.5%
79.9%
k41
k51
k61
k64
1-2 contigs
3-4 contigs
5-10 contigs
TOTAL
B
Percentage of contigs that represent >80% of chr 3B CDS
80.0%
70.0%
60.0%
50.0%
40.0%
30.0%
20.0%
10.0%
0.0%
63.5%
72.1%
72.6%
73.1%
k41
k51
k61
k64
1-2 contigs
3-4 contigs
5-10 contigs
TOTAL
